# Quercetin upregulates TLE4 to block the Wnt/β-catenin signaling pathway and inhibit cell migration and invasion of cervical cancer cells

**DOI:** 10.3389/fphar.2026.1892382

**Published:** 2026-10-07

**Authors:** Yirui Mai, Keyi Ju, Yongkang Yang, Weijing Wang, Ting Zhang, Jiake Wang, Yu Wang, Weihua Gao, Tuanwang Li, Beier Gong, Yuanyuan Li

**Affiliations:** 1 The Second Clinical Medical College, Shaanxi University of Chinese Medicine, Xianyang, Shaanxi, China; 2 The Second Affiliated Hospital, Shaanxi University of Chinese Medicine, Xianyang, Shaanxi, China; 3 The First Clinical Medical College, Yanan University, Yanan City, Shaanxi, China; 4 Institute of Integrated Traditional Chinese and Western Medicine for Oncology of the Second Affiliated Hospital, Shaanxi University of Chinese Medicine, Xianyang, Shaanxi, China

**Keywords:** cell invasion, cell migration, quercetin, TLE4, Wnt/β-catenin signaling pathway

## Abstract

**Methods:**

Using established TLE4-overexpression and CRISPR-Cas9-knockout cervical cancer cell lines and nude mouse tumorigenesis models, we verified the biological function and regulatory mechanism of TLE4 in cervical cancer.

**Results:**

Quercetin treatment upregulated the expression of TLE4 in HeLa cells. Compared with normal cervical tissues from clinical specimens, the expression of TLE4 in the nucleus of cervical cancer tissues was significantly downregulated. Transwell assays showed that the migration and invasion of C-33A TLE4-overexpression cells were significantly suppressed, accompanied by an upregulation of the expression of E-cadherin and a downregulation of the expression of N-cadherin, Vimentin, and Snail. Conversely, the migration and invasion of Ca Ski TLE4-knockout cells were significantly enhanced, and the expressions of these EMT-related markers showed the opposite trend. Meanwhile, the *in vivo* xenograft experiment revealed that the Ca Ski TLE4-knockout group exhibited faster tumor growth compared with the wild-type group. Furthermore, we examined the role of TLE4 in the Wnt/β-catenin signaling pathway. In C-33A TLE4-overexpression cells, β-catenin, TCF-1, TCF-4, CyclinD1, c-Myc, and MMP-7 were decreased, p-β-catenin expression was increased, whereas in Ca Ski TLE4-knockout cells, the expression trends of these factors were opposite. After treating TLE4-knockout Ca Ski cells with quercetin, the enhanced migration and invasion capabilities induced by TLE4 knockout were effectively reversed.

**Conclusion:**

Quercetin targets and upregulates the expression of TLE4, which inhibits the migration and invasion of cervical cancer cells by blocking the Wnt/β-catenin signaling pathway, thereby providing theoretical and experimental evidence for quercetin therapy in cervical cancer.

## 1 Introduction

Cervical cancer (CC) is a malignant tumor that poses a serious threat to women’s health. It is the fourth leading cause of morbidity and mortality of female malignant tumors in the world (Bray et al., 2024). Although great progress has been made in regular screening, vaccination of human papillomavirus (HPV), traditional surgery, chemoradiotherapy, immunotherapy and adjuvant therapy (Guimarães et al., 2022; Perkins et al., 2023; Zhao et al., 2025), morbidity and disease-specific mortality continue to rise in China (Han et al., 2024). The overall prognosis remains poor for patients with locally advanced, recurrent, or metastatic disease (Cohen et al., 2019; Li et al., 2025). Quercetin is a naturally occurring flavonol widely distributed in fruits, vegetables, and medicinal plants (Anand David et al., 2016; Chen et al., 2022; Li et al., 2016). Quercetin has shown anti-tumor effects in many tumors, such as breast cancer (Wu et al., 2025), colorectal cancer (Mehrabadi, 2025) and cervical cancer (Jian et al., 2024), by changing cell cycle, inhibiting cell proliferation, promoting cell apoptosis, inhibiting angiogenesis and metastasis, and affecting autophagy (Tang et al., 2020). Moreover, quercetin exerts anti-tumor effects partly through the modulation of microRNAs (Kim et al., 2019) and has shown protective effects in pulmonary fibrosis and its comorbidities (Vásquez-Garzón et al., 2025).

We previously treated HeLa cells with quercetin, and then carried out RNA-sequencing. Combining the NCBI GEO database with RNA-seq analysis, we identified the upregulated/downregulated genes in cervical cancer compared with normal cervix in the GEO database, and those that were reversely expressed after quercetin treatment. These genes may serve as potential therapeutic targets of quercetin (Li et al., 2021). We found that the expression of Transducin-like enhancer of split 4 (TLE4) is significantly decreased in cervical cancer compared with normal cervical tissue, while it is significantly upregulated after quercetin treatment. This suggests that TLE4 may play a tumor-suppressive role in the occurrence and development of cervical cancer. Additionally, quercetin can block the MAPK, PI3K, and Wnt signaling pathways (Kedhari Sundaram et al., 2019).

The human TLE family proteins consist of seven members (TLE1-7), of which TLE1-4 and TLE6 are full-length TLE family members, while TLE5/AES and TLE7 are truncated members. The full-length TLE protein has five domains, including the Q domain, GP domain, CcN domain, SP domain and WD repeat domain (Turki-Judeh and Courey, 2012). The TLE4 gene is located on chromosome 9q21.31, encoding 28 exons. The protein consists of 773 amino acids and has a relative molecular weight of 83.8 kDa. This protein is expressed in both the cytoplasm and nucleus. TLE4 protein is widely expressed in tissues, and its basic function is related to embryonic development (Galazo et al., 2023). Recently, it has been found that TLE4 plays an important role in cancer. It has been demonstrated that TLE4 exerts distinct effects in different tumors, for instance, it acts as a tumor suppressor in leukemia (Herold et al., 2017; Shin et al., 2016), liver cancer (Wu et al., 2016); whereas in colorectal cancer, TLE4 promotes the progression of the tumor (Wang et al., 2016). In addition, TLE4 plays a role in tumor drug resistance (Park et al., 2019; Wei et al., 2016), immune-inflammatory regulation (Shin et al., 2016; Zhang et al., 2019), prognosis (Cho et al., 2016) and targeted therapy (Prabhakaran et al., 2018; Wang et al., 2021). However, the function of TLE4 in cervical cancer and its underlying mechanism remain unclear.

In this study, we explored the biological functions and molecular mechanisms of quercetin-mediated upregulation of TLE4 in cervical cancer across three levels: clinical specimens, *in vitro* cell and *in vivo* animal experiments. Through the model of “active ingredient (quercetin) - target gene - pathway”, this study explores the molecular mechanism by which quercetin targets and upregulates TLE4 to block the Wnt/β-catenin signaling pathway, thereby inhibiting cervical cancer cell migration and invasion. This research provides theoretical and experimental evidence for the therapeutic application of quercetin in cervical cancer treatment.

## 2 Materials and methods

### 2.1 Cell lines and clinical samples

Human cervical epithelial H8 and cervical carcinoma cell lines HeLa, SiHa, and C-33A were purchased from Beijing Baiou Bowei Biotechnology Co., Ltd. Human cervical carcinoma cell lines HT-3 and Ca Ski were purchased from the American Type Culture Collection (United States). H8, HeLa, SiHa and C-33A cells were cultured in Dulbecco’s Modified Eagle’s Medium–high glucose (BasalMedia Technologies, China) supplemented with 10% fetal bovine serum (FBS, HyClone) and 1% penicillin–streptomycin solution. HT-3 and Ca Ski cells were cultured in McCoy’s 5A and RPMI-1640 (BasalMedia Technologies, China), respectively, supplemented with 10% FBS and 1% penicillin–streptomycin solution. All cell lines were incubated at 37 °C in an atmosphere containing 5% CO_2_.

The tissue microarray (F080Ce01) contained 80 tissue samples, including 40 each of normal and cancerous cervical tissue samples, purchased from Xi’an Zhongke Guanghua Bioaitech Co., Ltd. Sixteen additional cervical tissues (including eight each of normal and cancerous cervical tissue samples) were obtained from the Second Affiliated Hospital, Shaanxi University of Chinese Medicine. This study was approved by the Hospital Ethics Committee (GKJ-SQ-2021004). Informed consent has been obtained from all participants and their legal guardians in this study. All experimental procedures were performed in strict accordance with relevant guidelines and regulations. This study adhered to the ethical principles established by the Helsinki Declaration.

### 2.2 Immunohistochemistry

For immunohistochemical staining, the sections were subjected to baking, dewaxing, hydration, washing with distilled water, high-pressure antigen retrieval, washing with phosphate-buffered saline (PBS), encircling of the tissue area, endogenous peroxidase inactivation, and washing. Then, the sections were incubated with the primary antibody at 4 °C overnight, washed, incubated with the secondary antibody at room temperature, and washed again. Then, the samples were subjected to DAB chromogenic reaction, hematoxylin counterstaining, dehydration, transparency, sealing, and microscopic observation, followed by scanning. The following antibodies were used: TLE4 (1:200, ab140485, Abcam; 1:100, PA5-40533, Thermo Fisher Scientific). The grading criteria based on the proportion of positive cells were as follows: <5% (score 0); 6%–25% (score 1); 26%–50% (score 2); 51%–75% (score 3); ≥75% (score 4). The grading criteria for staining intensity were as follows: negative (score 0), weakly positive (score 1), moderately positive (score 2), and strongly positive (score 3). The final score was calculated using the following formula: Immunoreactivity score (IRS) = Score of positive cell percentage × Score of staining intensity.

### 2.3 Western blot analysis

Protein lysis buffer was prepared by adding an appropriate amount of Protease Inhibitor Cocktail (Roche) to RIPA lysis buffer (Thermo Fisher Scientific) at a ratio of 50:1. An appropriate amount of protein lysis buffer was added to the cells, which were then lysed on ice for 30–45 min. After centrifugation at 13,000 rpm and 4 °C for 15 min, the supernatant was collected. Protein quantification was performed using a bicinchoninic acid BCA kit (23,227, Thermo Fisher Scientific) according to the manufacturer’s instructions. After adding 5 × loading buffer, the mixture was incubated in boiling water for 5 min to denature the protein. After cooling, the samples were ready for use and could be stored at −80 °C for long-term preservation. Gel preparation was performed, followed by sample loading and sodium dodecyl sulfate-polyacrylamide gel electrophoresis. The proteins were then wet-transferred to a polyvinylidene fluoride membrane, which was blocked with 5% skimmed milk for 1.5 h, and incubated with primary antibodies at 4 °C overnight. The antibodies used were as follows: TLE4 (1:500, sc-365406, Santa Cruz; 1:500, Abcam, ab64833; 1:500, PA5-40533 and PA5-99730, Thermo Fisher Scientific), E-cadherin (1:2000, YT1454, Immunoway), N-cadherin (1:2000, YM8097, Immunoway), Vimentin (1:2000, YT4880, Immunoway), Snail (1:500, sc-271977, Santa Cruz), β-catenin (1:2000, YM8174, Immunoway), β-catenin (Phospho Thr41/Ser45) (1:2000, YM8446, Immunoway), Wnt-3a (1:1000, YM1459, Immunoway), TCF-1 (1:500, sc-271453, Santa Cruz), TCF-4 (1:2000, YN5827, Immunoway), CyclinD1 (1:2000, YM8149, Immunoway), c-Myc (1:2000, YT0991, Immunoway), MMP-7 (1:500, sc-80205, Santa Cruz), Histone H3 (1:2000, 4620, Cell Signaling Technology), GAPDH (1:1000, sc-465062, Santa Cruz). The membrane was washed with Tris-buffered saline with Tween 20 (TBST) and then incubated with horseradish peroxidase–conjugated secondary antibody at room temperature for 1.5 h. The secondary antibodies used were as follows: Goat anti-Rabbit IgG (H + L) Secondary Antibody (1:10,000, 31,460, Invitrogen) and Goat anti-Mouse IgG (H + L) Secondary Antibody (1:10,000, 31,430, Invitrogen). After washing the membrane with TBST, ECL chemiluminescent substrate was added for visualization using a gel imaging system. Semi-quantitative analysis was then performed using Quantity One software.

### 2.4 Real-time polymerase chain reaction

After designing the primers, total RNA was extracted from cells using the Trizol method. cDNA was synthesized by reverse transcription according to the instructions of the RevertAid RT Kit (K1691, Thermo Fisher Scientific), followed by quantification. The reaction system was prepared according to the instructions of SYBR™ Green PCR Master Mix (Thermo Fisher Scientific). The primer sequences were designed as follows: TLE1F (5′-3′)-GAG​CCG​GGC​ACA​AGT​AAT​TC; and TLE1R (5′-3′)-TCA​TTG​GAA​AAT​TCA​GGT​CCA​TT; TLE2F (5′-3′)-GCG​CCA​GCT​GCA​TTG​ATA​TT; and TLE2R (5′-3′)-CAG​TCC​TGG​TTA​GGG​CAG​TG; TLE3F (5′-3′)-ACG​TCT​ACA​CAG​GTG​GCA​AG; and TLE3R (5′-3′)-TTG​TCC​CTG​TTC​AGG​CAG​TC; TLE4F (5′-3′)-TGC​ATT​GTA​TTG​ACC​ACC​AGT; and TLE4R (5′-3′)-TAG​GAG​TGG​CAC​AGC​CAA​AG; AESF (5′-3′)-CAA​AGC​AGG​CAT​TCG​GGC; and AESR (5′-3′)-ACA​TCA​CAT​AGT​GAC​GCT​GC; TLE6F (5′-3′)-TCT​CCT​CAT​TTT​GCT​GCG​GA; and TLE6R (5′-3′)-CCT​CAG​ACT​GGA​AAC​CCT​GG; TLE7F (5′-3′)-GGG​CTT​ATG​GTG​AGC​CAG​AG; and TLE7R (5′-3′)-AGC​AGG​GCT​GTG​CTG​TAT​TG. The samples were detected using a QuantStudio™ five real-time fluorescent quantitative PCR instrument, followed by statistical analysis of the data.

### 2.5 Construction of TLE4 overexpression plasmid vectors and establishment of stable TLE4-overexpressing cells

Transcript information for TLE4 was retrieved using the NCBI online tool BLAST. Primers were designed using Premier 5.0 software. Total RNA was extracted from cells, followed by reverse transcription and PCR amplification. Subsequently, restriction enzyme digestion, gel extraction, and ligation using T4 DNA ligase were performed. The coding sequence region of the target gene was ligated into the pIRESpuro3 vector, and the vector map is presented in Supplementary Figure S2A. DH5α competent cells were transformed, and after recovery culture, the cells were plated on agar plates. After an overnight culture at 37 °C for 12–15 h, single clones were selected and sequenced to validate whether the sequence was correctly connected to the target vector (Supplementary Figure S2B).

TLE4 overexpression plasmid DNA was transfected into C-33A cells using Lipofectamine™ 3000 (L3000001, Thermo Fisher Scientific) according to the manufacturer’s instructions. After 24 h, 0.5 μg/mL of puromycin was added to the cells, which were then incubated for 48 h. After trypsinization, the cells were diluted and seeded into 10-cm culture dishes. Thereafter, single colonies were selected for further cultivation. Clones with stable TLE4 overexpression were identified via western blotting, after which they were labeled and preserved.

### 2.6 Construction of TLE4 knockout plasmid vectors using CRISPR/Cas9 technology

Transcript information of TLE4 was retrieved from the NCBI database, and the longer transcript with high homology was selected as the knockout target. Based on its corresponding amino acid sequence, analysis using the online NCBI CD-search tool revealed that the active region of this transcript product contains the Q domain and the WD repeat domain (Supplementary Figure S3A). We selected exon 2, which is included in all transcripts, as the region for designing the knockout site (Supplementary Figure S3B).

Knockout sites were designed using online software, with the following three sites: KO1, AAAGTCAATATCGTTACCTG; KO2, CGAATCCTGTGATCGGATTA; and KO3, TTTTACAGGCTCAATACCAC. The specific positions are shown in Supplementary Figure S3C. Oligo primers were designed and synthesized. The single guide RNA in the vector was driven by the U6 promoter, which requires the first base to be “g.” Thus, the oligo sequences were designed as follows:

H-TLE4-KO1F: CAC​CgA​AAG​TCA​ATA​TCG​TTA​CCT​G
H-TLE4-KO1R: AAA​CCA​GGT​AAC​GAT​ATT​GAC​TTT​c
H-TLE4-KO2F: CAC​CgC​GAA​TCC​TGT​GAT​CGG​ATT​A
H-TLE4-KO2R: AAA​CTA​ATC​CGA​TCA​CAG​GAT​TCG​c
H-TLE4-KO3F: CAC​CgT​TTT​ACA​GGC​TCA​ATA​CCA​C
H-TLE4-KO3R: AAA​CGT​GGT​ATT​GAG​CCT​GTA​AAA​c

The knockout vector was constructed, and the hybridization system was prepared. The mixture was incubated at 98 °C for 3 min in a PCR instrument and then cooled down at a rate of 1 °C per minute until room temperature. A ligation system was prepared and incubated at room temperature for approximately 1 h. Subsequently, DH5α competent cells were transformed, plated on agar plates, and cultured overnight at 37 °C. Single colonies were selected for sequence identification. A map of the successfully constructed vector (pCas-Puro-U6-H-TLE4-KO) is shown in Supplementary Figure S3D. The bacterial culture was stored at −80 °C, and the plasmid could be directly used for cell transfection.

### 2.7 Establishment of stable TLE4-knockout cells

Using Lipofectamine™ 3000 transfection reagent, the plasmid vectors (TLE4-KO1, TLE4-KO2, TLE4-KO3, KO1/KO2, and KO2/KO3) were transfected into Ca Ski cells. After 24 h, 1.5 μg/mL of puromycin was added to the cells, which were then incubated for 48 h. After trypsinization, the cells were diluted and seeded into 10-cm culture dishes. Afterwards, single colonies were selected for further cultivation, and genomic DNA was rapidly extracted. PCR amplification was performed using the primers TLE4-cas-F (5′–3′)-TCC​CTG​GGT​GCC​TGT​CTT​TG and TLE4-cas-R (5′–3′)-GTT​TGG​CCT​CAG​CGT​TTT​CC. Thereafter, the PCR products were sent to Thermo Fisher Scientific for sequencing.

### 2.8 Transwell assay

Transwell assay with and without Matrigel was used to detect cell invasion and migration, respectively. For the Matrigel-coated Transwell assay, Matrigel (Corning) was diluted at a ratio of 1:3 to 1:8 on ice and added to the upper chambers of a 24-well plate. Thereafter, approximately 80 μL of Matrigel was evenly spread into the upper chambers and incubated at 37 °C for 4–5 h for later use. Logarithmically growing cells were harvested, trypsinized, washed sequentially with PBS and serum-free medium, and then resuspended in serum-free medium. After counting, the cell concentration was adjusted to 2 × 10^5^ cells/mL. Afterwards, 600 μL of complete medium was added to the lower chambers, and 100–150 μL of the cell suspension was added to the upper chambers, followed by routine culture. After 24 and 48 h of culture, the upper chambers were carefully removed, the culture medium was aspirated, and the cells were fixed with methanol at room temperature for 30 min. After aspirating the methanol, the cells were stained with crystal violet solution for 30 min. The cells were rinsed several times with purified water, and the excess liquid was aspirated. Cells on the upper surface of the membrane in the upper chamber were carefully wiped off with a moist cotton swab. The membrane was then fixed on a glass slide with its bottom surface facing up for photography, followed by manual cell counting and statistical analysis.

### 2.9 Tumor xenograft experiment

Female BALB/c nude mice aged 5–6 weeks were purchased from the Experimental Animal Center of Shaanxi University of Chinese Medicine and reared in the specific pathogen-free (SPF) animal center. Logarithmically growing cells were harvested, counted, and the cell concentration was adjusted to 2.5 × 10^7^ cells/mL. The cell suspension was kept on ice, and 200 μL of the suspension was subcutaneously injected into the back of the mice. Tumor size was recorded every 3 days, and tumor volume was calculated using the following formula: Volume = 0.5 × length × width^2^. Tumor growth was monitored continuously, and tumor growth curves were plotted. The mice were then euthanized in accordance with animal ethics guidelines and animal welfare regulations. Following induction of deep anesthesia via intraperitoneal injection of a 2% sodium pentobarbital solution (50 mg/kg), mice were euthanized with an overdose of the agent in accordance with AVMA guidelines. Tumor tissues were promptly excised and weighed. Tumor tissues were fixed in neutral formalin solution and embedded in paraffin.

For the quercetin-intervention experiment, female BALB/c nude mice aged 5–6 weeks were acclimated for 1 week, with food and water replenished every 3–4 days. Logarithmically growing SiHa cells were harvested, counted, and maintained on ice. The dorsal skin was disinfected with iodophor, and a cell suspension containing 4 × 10^6^ cells was subcutaneously injected using a 0.5 mL syringe. Mice were randomly assigned to four groups (n = 8 per group): vehicle control group, cisplatin group (2 mg/kg), low-dose quercetin group (50 mg/kg), and high-dose quercetin group (100 mg/kg). All treatments were administered via intraperitoneal injection once every 2 days, and tumor volumes were measured every 3 days. Upon completion of treatment, animals were euthanized according to the aforementioned anesthesia and euthanasia protocol. Tumor tissues were promptly excised, weighed and fixed in neutral formalin solution for subsequent analyses.

The animal experiments were approved by the Experimental Animal Ethics Committee of Shaanxi University of Chinese Medicine (SUCMDL20210305011). The study is reported in accordance with ARRIVE guidelines.

### 2.10 Source of drugs

Quercetin was purchased from MedChemExpress (HY-18085). Quercetin was dissolved in dimethyl sulfoxide (DMSO) to prepare a 600 mM stock solution, which was aliquoted and stored at −20 °C. Wnt/β-catenin agonist one was purchased from MedChemExpress (HY-114321). Wnt/β-catenin agonist one was dissolved in DMSO to prepare a 10 mM stock solution, which was aliquoted and stored at −20 °C. For the rescue experiments, cells were treated with the Wnt/β-catenin agonist 1 (3.5 μM), alone or in combination with quercetin (100 μM) for 48 h, followed by western blot analysis.

### 2.11 CCK-8

Cells in logarithmic growth phase were washed with PBS, digested with trypsin for 3–5 min, and neutralized with complete medium. The cell suspension was centrifuged at 1,000 rpm for 5 min. The supernatant was removed, and cells were resuspended in 1 mL complete medium. After counting using a hemocytometer, cells were seeded into 96-well plates at 5,000 cells/well in a final volume of 200 μL (2.5 × 10^4^ cells/mL) and incubated at 37 °C in 5% CO_2_ for 24 h to allow attachment. Control (DMSO-containing medium), blank (medium only), and quercetin treatment groups were set, each with six replicates. The 600 mM quercetin stock solution was diluted to 200 μM as the highest working concentration and then serially diluted two-fold to yield 100, 50, 25, and 12.5 μM. The old medium was replaced with 200 μL of drug-containing medium, and cells were incubated for 24, 48, and 72 h. A 10% CCK-8 working solution was freshly prepared by diluting the CCK-8 stock solution 1:9 with complete medium. At each time point, the drug-containing medium was removed, and 200 μL of CCK-8 working solution was added to each well. The plate was incubated for 1–4 h at 37 °C in 5% CO_2_. Absorbance was measured at 450 nm. Cell viability was calculated as: *Cell viability (%) = [OD(treatment) – OD(blank)]/[OD(control) – OD(blank)] × 100%.*

### 2.12 Co-immunoprecipitation (Co-IP) assay

Co-IP was performed using the Pierce Co-IP Kit (88,804, Thermo Fisher Scientific) according to the manufacturer’s instructions. Cells were harvested at 70%–80% confluence and lysed with ice-cold IP lysis/wash buffer on ice for 30 min with intermittent vortexing. After centrifugation, the supernatant was collected, and quantified using the BCA assay. To immobilize antibodies on AminoLink resin, 50 μL of anti-TLE4 antibody (sc-365406, Santa Cruz) or IgG (sc-515946, Santa Cruz) was coupled to AminoLink resin with gentle rotation at room temperature for 2 h. Equal amounts of protein (500 μg) from each cell lysate were incubated with the antibody-coupled resin overnight at 4 °C with gentle rotation. After extensive washing, bound proteins were eluted with elution buffer, mixed with 5 × loading buffer, heated at 95 °C for 10 min, and detected by western blot analysis.

### 2.13 Nuclear-cytoplasmic fractionation

Nuclear and cytoplasmic proteins were extracted using a commercial kit (P0028, Beyotime) per the manufacturer’s protocol. Cells were harvested, washed with ice-cold PBS, and centrifuged. The pellet was resuspended in cytoplasmic extraction reagent A supplemented with 1 mM PMSF, vigorously vortexed, and incubated on ice for 15 min. Reagent B was then added, and the mixture was vortexed and incubated on ice for 1 min. After centrifugation at 12,000 × g for 5 min at 4 °C, the supernatant (cytoplasmic fraction) was collected. The nuclear pellet was resuspended in nuclear extraction reagent supplemented with 1 mM PMSF, vortexed, and incubated on ice for 30 min with intermittent vortexing. After centrifugation at 12,000 × g for 10 min at 4 °C, the supernatant (nuclear fraction) was collected. Proteins were quantified using the BCA assay.

### 2.14 Statistical analysis

Statistical analyses were performed using the GraphPad Prism V10.0 software (La Jolla, United States). Univariate analysis was performed using Student’s t-test (two groups) or one-way ANOVA (three or more groups). Two-way ANOVA was used for two-factor comparisons. Multiple-comparison corrections were performed with Tukey’s multiple comparisons test. Data were presented as mean ± standard error of the mean (SEM). In all tests, a p value of <0.05 indicated statistical significance.

## 3 Results

### 3.1 Quercetin regulates the expression of TLE family genes

Analysis of the expression of the TLE family genes across three microarray datasets in the GEO database (GSE7803, GSE9750, and GSE63514) revealed a downregulation of TLE4 in cervical cancer (Figure 1A). After treating HeLa cells with quercetin, RNA-seq results showed an upregulation of TLE1, TLE2, TLE4, and TLE6 and a downregulation of AES/TLE5 (Figure 1B). We then screened out genes that showed increased/decreased expression in cervical cancer compared with normal cervical tissue in the GEO database, and exhibited reversed expression trends after quercetin treatment. Through this approach, TLE1, TLE4, and TLE6 were identified as potential targeted therapeutic genes. RNA expression levels of the TLE family genes were further examined via real-time polymerase chain reaction (PCR). Notably, we found that TLE4 expression was most prominently altered (Figure 1C). Therefore, we focused on TLE4 as a target gene and explored its role in the progression of cervical cancer and underlying mechanisms.

**FIGURE 1:**
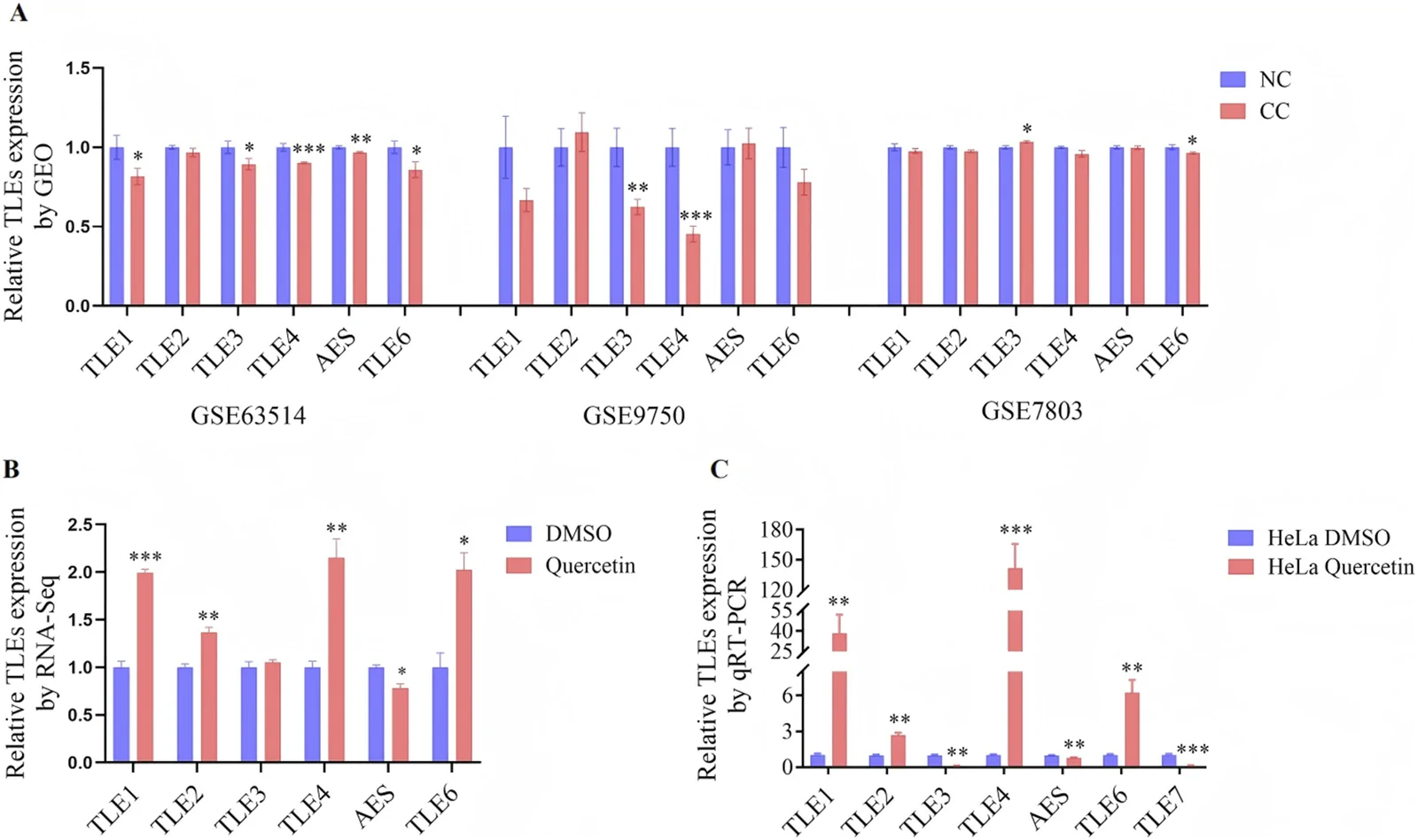
Combined GEO database and RNA-seq analysis identified TLE4 as an important target gene of quercetin in cervical cancer. **(A)** Expression of TLE family genes in three microarray datasets from the GEO database (GSE7803, GSE9750, and GSE63514). **(B)** Expression of TLE family genes based on RNA-seq results after quercetin treatment. **(C)** Real-time PCR was used to validate the mRNA expression of the TLE family genes in HeLa cells after quercetin treatment. RNA-seq, RNA-sequencing; TLE4, Transducin-like enhancer of split 4; PCR, polymerase chain reaction. Significant differences are indicated by *p < 0.05, **p < 0.01, ***p < 0.001.

### 3.2 TLE4 expression in cervical cancer tissues

TLE4 expression in clinical cervical cancer tissue microarray (including 40 normal and 40 cancerous cervical tissue samples) was detected through immunohistochemistry. Analysis of TLE4 expression in the cytoplasm and cell nucleus revealed that compared with the normal cervical tissues, TLE4 expression was significantly higher in the cytoplasm and lower in the nucleus of cervical cancer tissues, respectively. Overall, TLE4 expression was significantly lower in cervical cancer tissues than in normal cervical tissues (Figures 2A,B; Supplementary Figure S1). Real-time PCR was further used to verify TLE4 expression at the RNA level in cervical tissues (eight samples each from both groups), which showed that TLE4 expression was significantly lower in cervical cancer tissues than in normal cervical tissue (Figure 2C). No significant correlation was observed between TLE4 expression and patient age, tumor stage, or lymph node metastasis based on clinicopathological analysis.

**FIGURE 2:**
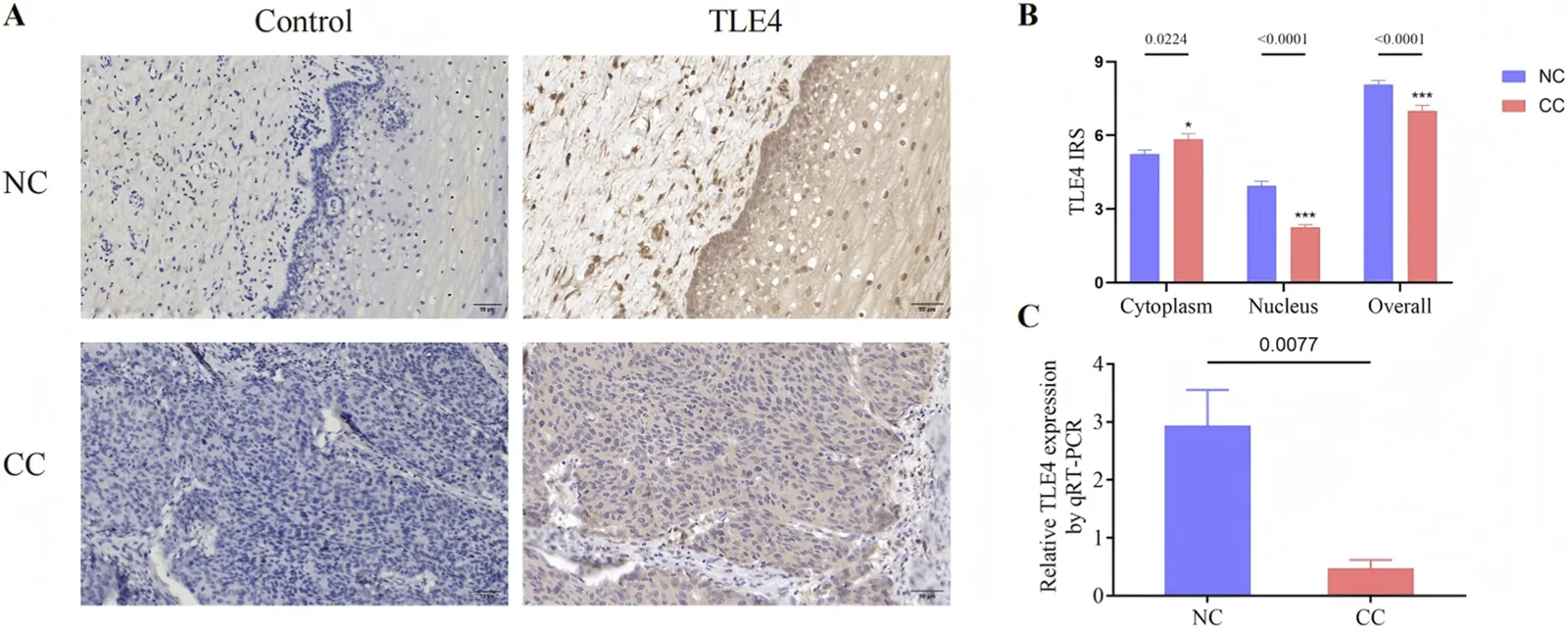
Expression of TLE4 in clinical cervical tissues. **(A)** Immunohistochemical staining of TLE4 in NC (n = 40) and CC (n = 40) tissues. PBS, in place of the primary antibody, served as the blank control. (magnification: 40×, scale bar = 50 μm). **(B)** Statistical analysis of TLE4 immunoreactivity scores between NC and CC shown in **(A)**. **(C)** Detection of TLE]4 expression in cervical tissues using real-time PCR (n = 8 per group). *p < 0.05, ***p < 0.001 indicate statistically significant differences. TLE4, Transducin-like enhancer of split 4; NC, normal human cervical tissue; CC, cervical cancer tissue.

### 3.3 TLE4 expression in cervical cancer cell lines and establishment of TLE4 expression regulatory cells

Western blot analysis was used to detect TLE4 expression in cervical epithelial H8, cervical cancer HeLa, SiHa, C-33A, HT-3, and Ca Ski cell lines. As shown in Figure 3A, TLE4 expression was low in H8, SiHa, C-33A, and HT-3 cells but high in HeLa and Ca Ski cells. Therefore, we constructed a TLE4 overexpression plasmid vector (Supplementary Figure S2) and transfected it into C-33A cells to obtain stable C-33A TLE4-overexpressing cell lines. Successful TLE4 overexpression was confirmed by western blot analysis (Figure 3C). The TLE4 knockout plasmid vector (Supplementary Figure S3) was constructed using CRISPR-Cas9 technology. It was transfected into Ca Ski cells to obtain stable TLE4 knockout Ca Ski cells. The TLE4 expression levels in both cell groups were validated by western blot and DNA sequencing (Figures 3D–F, respectively).

**FIGURE 3:**
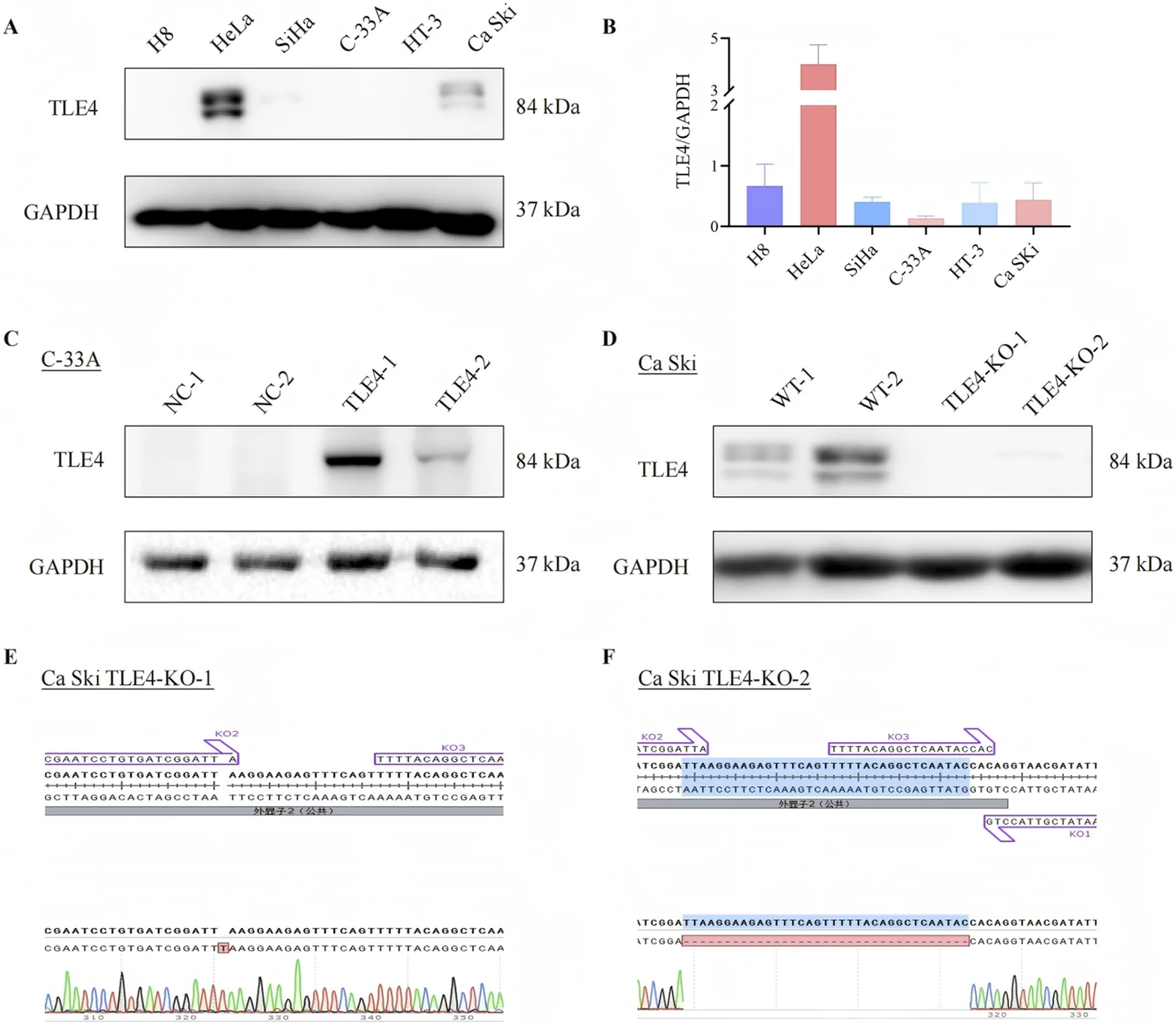
TLE4 expression in cervical cancer cell lines and establishment of TLE4 expression regulatory cells. **(A)** TLE4 expression in cervical epithelial cell line H8 and cervical cancer cell lines HeLa, SiHa, C-33A, HT-3, and Ca Ski. **(B)** Quantitative analysis of **(A)**. Data are shown as mean ± SEM (n = 3). **(C)** Detection of TLE4 protein expression in C-33A NC and TLE4-overexpressing cells via western blot analysis. **(D)** Detection of TLE4 protein expression in WT and TLE4-KO Ca Ski cells via western blot analysis. **(E)** Sequencing results of TLE4-KO-1 Ca Ski cells with a 1-bp insertion. **(F)** Sequencing results of TLE4-KO-2 Ca Ski cells with a 40-bp deletion. TLE4, Transducin-like enhancer of split 4; NC, normal control; WT, wild-type; KO, knockout.

### 3.4 TLE4 inhibits the migration and invasion of cervical cancer cells

Transwell assay was used to detect the effects of TLE4 overexpression or knockdown on the migration and invasion of cervical cancer cells. Our results showed that in C-33A cells, TLE4 overexpression significantly suppressed cell migration and invasion (Figures 4A,B) while simultaneously increasing E-cadherin expression and decreasing the expression of N-cadherin, Vimentin, and Snail (Figures 4E,F). In Ca Ski cells, TLE4 knockout significantly enhanced the cell migration and invasion (Figures 4C,D) while concurrently decreasing E-cadherin expression and increasing the expression of N-cadherin, Vimentin, and Snail (Figures 4G,H).

**FIGURE 4:**
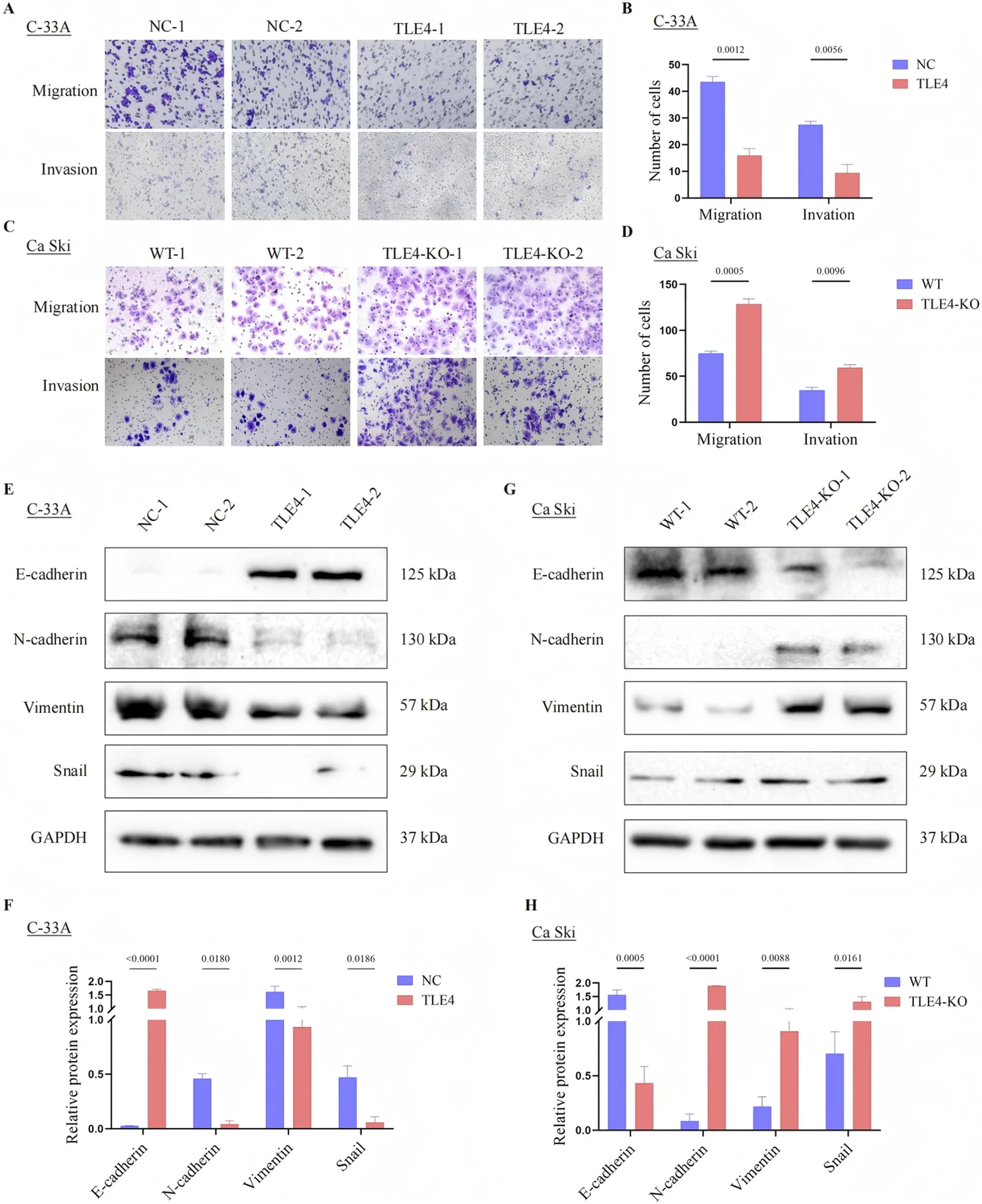
TLE4 inhibits the migration and invasion abilities of cervical cancer cells. **(A)** Migration and invasion were assessed in C-33A NC and TLE4-overexpressing cells using the Transwell assay. **(B)** Statistical analysis of panel A. **(C)** Migration and invasion were assessed in Ca Ski WT and TLE4-KO cells using the Transwell assay. **(D)** Statistical analysis of panel C. **(E)** Protein expression of migration- and invasion-related factors in C-33A NC and TLE4-overexpressing cells. **(F)** Statistical analysis of panel E. **(G)** Protein expression of migration- and invasion-related factors in Ca Ski WT and TLE4-KO cells. **(H)** Statistical analysis of **(G)**. TLE4, Transducin-like enhancer of split 4; NC, normal control; WT, wild-type; KO, knockout.

### 3.5 TLE4 knockout promotes the growth of xenograft tumors in nude mice

An immunodeficient nude mouse xenograft tumor model was used to evaluate the role of TLE4 in tumorigenesis *in vivo*. Wild-type and TLE4-knockout Ca Ski cells were subcutaneously inoculated into nude mice (Figure 5A). The xenograft tumor images, growth curve, and tumor weight are shown in Figures 5B–D, respectively. The growth curve showed that the TLE4-knockout Ca Ski cells had markedly increased tumor growth and a significantly faster growth rate and larger final volume than the wild-type cells (p < 0.05).

**FIGURE 5:**
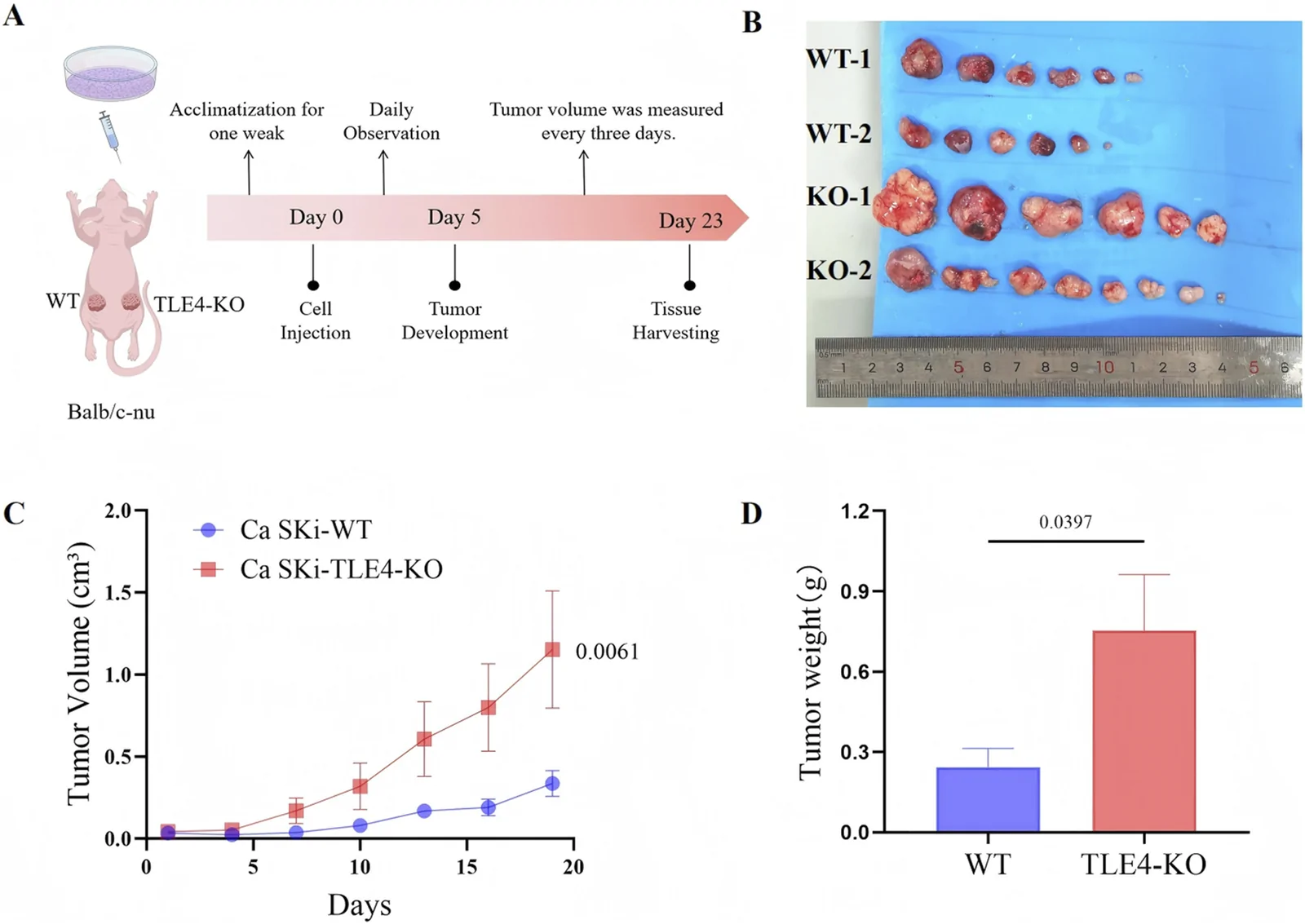
Tumorigenesis experiment in nude mice. **(A)** Flow chart of xenograft tumor formation in nude mice. **(B)** Images of transplanted tumors in nude mice (WT group vs. TLE4-KO group). **(C)** Tumor growth curves in the WT and TLE4-KO groups. **(D)** Assessment of xenograft tumor weight in tissues harvested at the experimental endpoint. TLE4, Transducin-like enhancer of split 4; WT, wild-type; KO, knockout.

### 3.6 Quercetin inhibits the Wnt/β-catenin signaling pathway by upregulating TLE4

Analysis of Gene Ontology functions, Kyoto Encyclopedia of Genes and Genomes (KEGG) pathway enrichment, and protein–protein interactions of TLE4 revealed that TLE4 is involved in transcriptional regulation. It can form a complex with β-catenin-T-cell factors (TCFs) and participate in the Wnt/β-catenin signaling pathway. TLE4 also interacts with Wnt pathway-related proteins, such as β-catenin, and TCF family proteins, including TCF1, LEF1, TCF3 and TCF4 (also known as TCF7, LEF1, TCF7L1 and TCF7L2, respectively; Supplementary Figure S5).

To explore whether TLE4 regulates the Wnt/β-catenin signaling pathway, we assessed the expression of Wnt signaling pathway-related factors and downstream proteins in TLE4-overexpressing and TLE4-knockout cells. Our results showed that in C-33A cells, TLE4 overexpression decreased the expression of Wnt-3a, β-catenin, TCF-1, TCF-4 and downstream proteins (CyclinD1, c-Myc, and MMP-7) while concurrently increasing the expression of p-β-catenin (Figures 6A,B). However, TLE4 knockout promoted an opposite expression trend in Ca Ski cells (Figures 6C,D). Furthermore, after treating HeLa cells with quercetin, we used the co-immunoprecipitation (Co-IP) method to precipitate and enrich TLE4-binding proteins. The results of our western blot analysis showed that in the control group, TLE4 bound to Wnt-3a and β-catenin; however, it no longer interacted with them after quercetin treatment. In the quercetin group, among the total input proteins, TLE4 expression increased, whereas the expression of Wnt-3a, β-catenin, TCF1, and TCF4 decreased (Figure 6E). Furthermore, nuclear-cytoplasmic fractionation showed that quercetin markedly decreased nuclear TLE4 while increasing its cytoplasmic level (Figure 6F), indicating that quercetin induces the nuclear export of TLE4 from the nucleus to the cytoplasm. Concurrently, both nuclear and total β-catenin levels were substantially decreased. To determine whether quercetin acts specifically through the Wnt/β-catenin pathway, we performed rescue experiments using the Wnt agonist one in Ca Ski cells (Figure 6G). Treatment with Wnt agonist one alone markedly upregulated β-catenin, Cyclin D1, and c-Myc, confirming activation of the Wnt pathway. Quercetin alone increased TLE4 protein levels and suppressed Wnt/β-catenin signaling. In the co-treatment group, although β-catenin levels were partially rescued compared with the quercetin-alone group, Cyclin D1 and c-Myc remained markedly suppressed. These results indicated that quercetin targets and upregulates TLE4, thereby inhibiting the Wnt/β-catenin signaling pathway.

**FIGURE 6:**
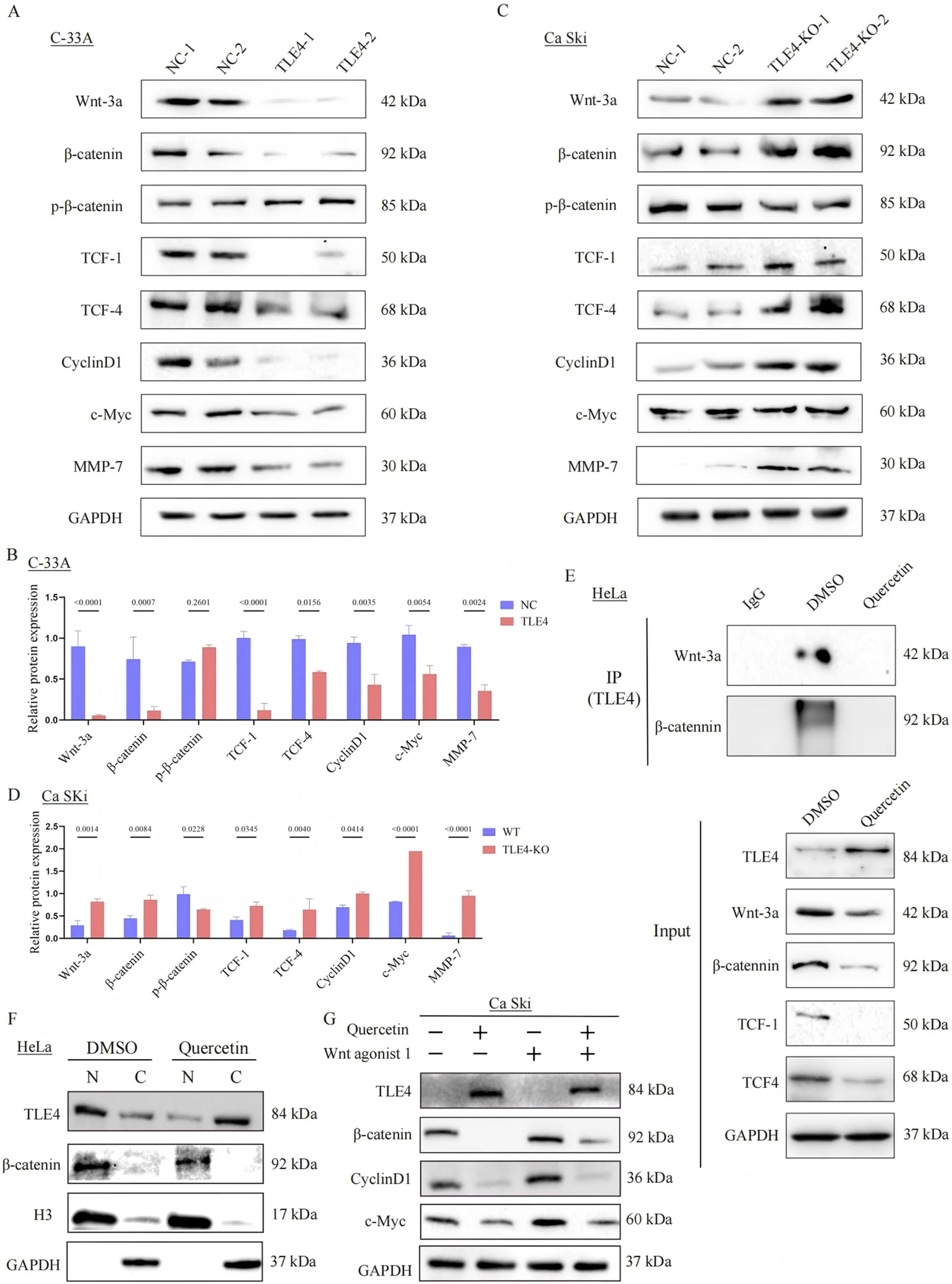
Quercetin targets and upregulates TLE4 to inhibit the Wnt/β-catenin signaling pathway. **(A)** Protein expression of Wnt/β-catenin signaling pathway proteins and their downstream target genes in C-33A NC and TLE4-overexpressing cells. **(B)** Statistical analysis of panel A. **(C)** Protein expression of Wnt/β-catenin signaling pathway proteins and their downstream target genes in Ca Ski WT and TLE4-KO cells. **(D)** Statistical analysis of panel C. **(E)** TLE4-binding proteins enriched by Co-IP and the protein expression of TLE4 and Wnt/β-catenin signaling pathway proteins in the control and quercetin groups. **(F)** Nuclear and cytoplasmic fractionation of TLE4 and β-catenin in HeLa cells treated with or without quercetin; **(G)** Protein expression of TLE4, β-catenin, Cyclin D1, and c-Myc in Ca Ski cells treated with the Wnt agonist 1 (3.5 μM) and/or quercetin (100 μM). TLE4, Transducin-like enhancer of split 4; NC, normal control; WT, wild-type; KO, knockout; Co-IP, co-immunoprecipitation.

### 3.7 Quercetin reverses TLE4 knockout-induced migration and invasion

In this study, the inhibitory effect of quercetin on the proliferation of cervical cancer Ca Ski cells was evaluated using the CCK-8 cell proliferation assay. The half-maximal inhibitory concentration (IC_50_) of quercetin at 24 h was determined to be 147.1 μM (Figure 7A), at 48 h was 90.74 μM (Figure 7B), and at 72 h was 140.2 μM (Figure 7C).

**FIGURE 7:**
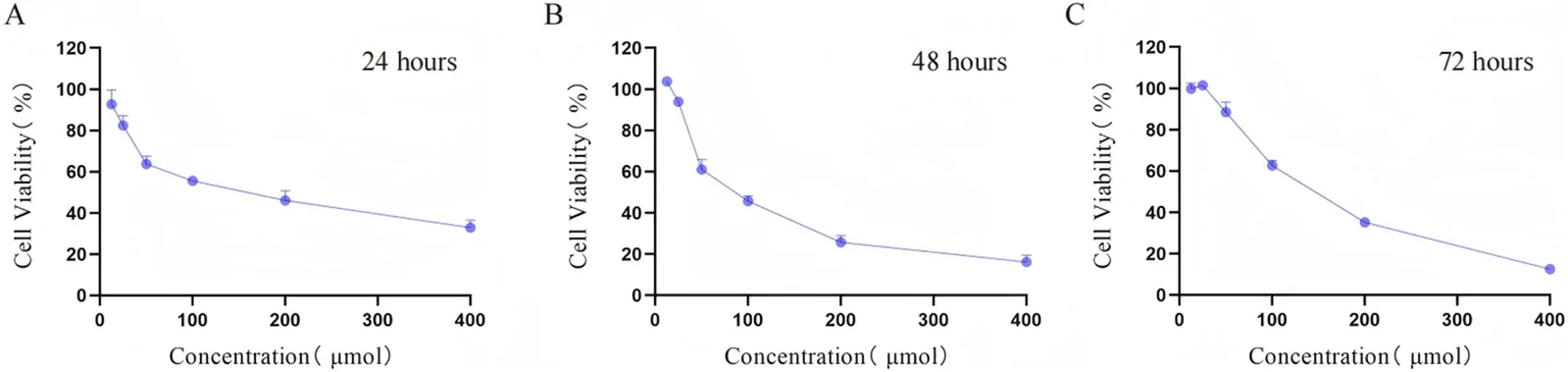
Quercetin inhibits the proliferation of Ca Ski cells. **(A)** Cell viability after 24 h of quercetin treatment. **(B)** Cell viability after 48 h of quercetin treatment. **(C)** Cell viability after 72 h of quercetin treatment.

We treated TLE4-knockout Ca Ski cells with quercetin. Transwell assay results showed that quercetin significantly reversed the enhanced cell migration and invasion induced by TLE4 knockout (Figures 8A,C). However, after quercetin treatment, the migration and invasion of TLE4-knockout cells remained higher than those of quercetin-treated wild-type cells, indicating that the inhibitory effect of quercetin is partially dependent on TLE4 (Figures 8B,D). Western blot analysis further demonstrated that quercetin treatment decreased N-cadherin expression and significantly downregulated the expression levels of key proteins of the Wnt/β-catenin signaling pathway, including Wnt-3a and β-catenin, as well as its downstream target genes CyclinD1 and c-Myc (Figures 8E,F).

**FIGURE 8:**
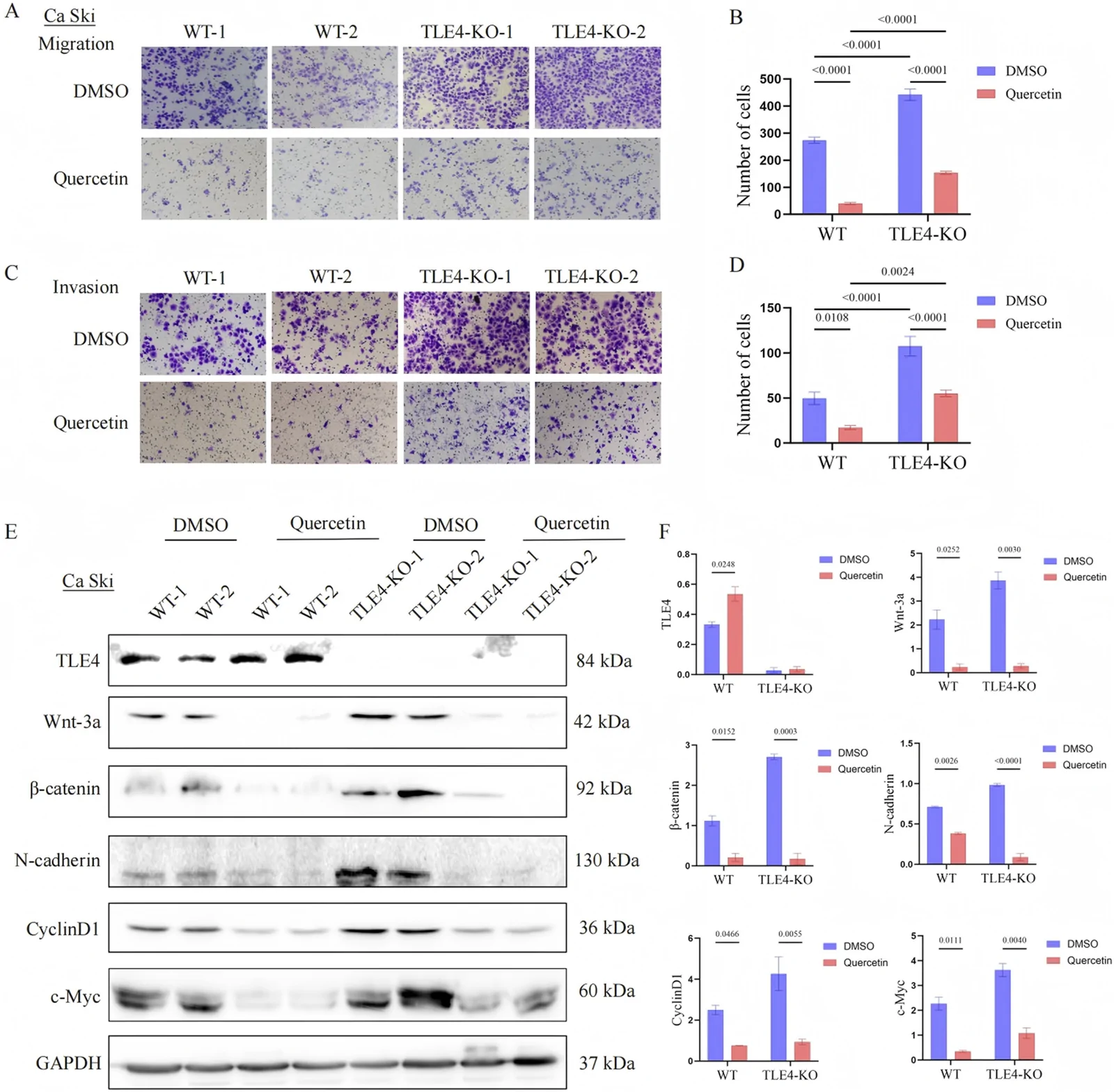
Quercetin reverses TLE4 knockout-induced migration and invasion. **(A)** Transwell migration assay of Ca Ski WT and TLE4-KO cells treated with DMSO or quercetin. **(B)** Statistical analysis of panel A. **(C)** Transwell invasion assay of Ca Ski WT and TLE4-KO cells treated with DMSO or quercetin. **(D)** Statistical analysis of panel C. **(E)** Protein expression of TLE4, Wnt-3a, β-catenin, N-cadherin, Cyclin D1, and c-Myc in Ca Ski WT and TLE4-KO cells treated with DMSO or quercetin. **(F)** Statistical analysis of **(E)**. TLE4, Transducin-like enhancer of split 4; WT, wild-type; KO, knockout.

## 4 Discussion

Cervical cancer patients treated with radiotherapy and chemotherapy often suffer from side effects. These include toxicity, untargeted tissue damage, hair loss, drug resistance, and nausea (Anand et al., 2023). Quercetin is a naturally occurring polyphenolic compound belonging to the flavonol subclass of flavonoids, which exerts effects in both the early and late stages of tumorigenesis, thereby preventing and inhibiting the development of cancer (Tang et al., 2020). At the same time, quercetin is a plant secondary metabolite that is often found in small amounts in fruits and vegetables. An appropriate amount of quercetin can be taken as a dietary supplement with few side effects (Andres et al., 2018). Quercetin exhibits favorable pharmacological activities, including anti-SARS-CoV-2, antioxidant, anticancer, anti-aging, antiviral, and anti-inflammatory activities (Wang et al., 2022). However, quercetin also acts on multiple targets and pathways in tumor suppression (Asgharian et al., 2022). The complexity of its mechanism of action prompted us to conduct the present study.

We first detected TLE4 expression in clinical cervical tissue specimens and separately quantified its cytoplasmic and nuclear levels in the normal cervical and cervical cancer groups. We found that the difference in TLE4 expression in the nucleus between the two groups was more pronounced. We used antibodies from Abcam, Santa Cruz, and Thermo Fisher Scientific, respectively, to ensure the accuracy of the experiment. Our results suggest that TLE4 may function as a tumor suppressor in the development of cervical cancer, and that TLE4 is a downstream target gene of quercetin. We constructed TLE4 overexpression and CRISPR/Cas9 knockout plasmid vectors, which were transfected into C-33A cells and Ca Ski cells, respectively, to establish stable TLE4-overexpression and TLE4-knockout cell lines. Transwell assay results showed that after stable overexpression of TLE4, the migration and invasion of C-33A cells were significantly decreased. Meanwhile, the expression of E-cadherin was increased, and the expressions of N-cadherin, Vimentin, and Snail were decreased. After knockdown of TLE4, the cell migration and invasion were significantly increased, and the expression trends of these factors were opposite (Figure 4). These results confirm that TLE4 inhibited the migration and invasion of cervical cancer cells.

Further verification was conducted using an *in vivo* tumor xenograft assay. The Ca Ski wild-type and TLE4-knockout cells were inoculated into nude mice. The results showed that, compared with the control group, the growth rate of xenograft tumors was accelerated in the TLE4-knockout group. We also conducted an *in vivo* tumorigenesis experiment using C-33A cells; however, no tumorigenesis was observed.

We also established a SiHa xenograft model and treated tumor-bearing mice with quercetin (50 or 100 mg/kg), with cisplatin as a positive control, to evaluate its *in vivo* anti-tumor activity. As shown in Supplementary Figure S4, quercetin treatment moderately inhibited tumor growth and reduced final tumor weight compared with the vehicle control, although the differences did not reach statistical significance. The relatively modest *in vivo* efficacy of quercetin, despite its potent anti-tumor activity *in vitro*, is likely attributable, at least in part, to its unfavorable pharmacokinetic properties. Quercetin exhibits low aqueous solubility and poor bioavailability and undergoes rapid metabolism and clearance, resulting in limited drug exposure in tumor tissues (Wang et al., 2022). Consequently, the local concentrations achieved *in vivo* may not recapitulate the effective concentrations used *in vitro*. In addition, quercetin acts as a multitarget, multipathway regulator. *In vitro*, high concentrations can simultaneously engage multiple cancer-relevant pathways, producing a strong cumulative effect. *In vivo*, however, the substantially lower drug exposure may only allow partial or transient modulation of each target, resulting in a net effect that is broad but limited.

Notably, although the *in vivo* tumor-suppressive effect of quercetin did not achieve statistical significance, the observed inhibitory trend was directionally consistent with the quercetin-induced upregulation of TLE4 and suppression of Wnt/β-catenin signaling observed *in vitro*. Together with the accelerated xenograft growth upon TLE4 knockout (Figure 5), these observations suggest that TLE4 may serve as a potential *in vivo* target of quercetin, whereas the poor pharmacokinetic profile of quercetin may restrict the full realization of its therapeutic efficacy *in vivo*.

To further evaluate the therapeutic potential of quercetin in cervical cancer, our ongoing and future studies will focus on improving its bioavailability and tumor accumulation through formulation strategies, including nanoformulations (e.g., PLGA nanoparticles, liposomes, or micelles), cyclodextrin inclusion complexes, or phospholipid complexes, as well as optimized dosing regimens, such as more frequent administration or continuous delivery (Nam et al., 2016). These approaches are expected to enhance drug exposure in tumor tissues, thereby enabling a more robust assessment of quercetin’s *in vivo* efficacy and its TLE4-mediated antitumor mechanism. In addition, we plan to investigate whether TLE4 expression levels in tumor tissues correlate with treatment response, which would further support TLE4 as a predictive biomarker of quercetin sensitivity.

Bioinformatics analysis showed that TLE4 could form a complex with β-catenin-TCFs and participate in the Wnt/β-catenin signaling pathway. Western blot analysis showed that in C-33A TLE4-overexpression cells, the expression levels of Wnt-3a, β-catenin, TCF1, TCF4, and downstream proteins (CyclinD1, c-Myc, and MMP-7) were decreased, while the expression of p-β-catenin was increased. In Ca Ski TLE4-knockout cells, the expression of Wnt/β-catenin pathway-related factors showed an opposite trend. The data indicate that TLE4 overexpression leads to inactivation of the Wnt/β-catenin signaling pathway, while its knockdown reverses this effect. Furthermore, we treated HeLa cells with quercetin and used the co-immunoprecipitation (Co-IP) assay to precipitate and enrich TLE4-binding proteins. Western blot results showed that TLE4 bound Wnt-3a and β-catenin in the control group. However, after quercetin treatment, TLE4 no longer bound with Wnt-3a and β-catenin. In the total proteins, the expression of TLE4 was significantly increased, while the expressions of Wnt-3a, β-catenin, TCF1, and TCF4 were decreased (Figure 6).

To address the apparent paradox that quercetin upregulates TLE4 yet reduces the TLE4–β-catenin interaction in Co-IP assays, we performed nuclear-cytoplasmic fractionation (Figure 6F). Quercetin induced the nuclear export of TLE4, as reflected by markedly decreased nuclear TLE4 and a corresponding increase in the cytoplasmic fraction. Concurrently, both nuclear and total β-catenin levels were substantially decreased. Given that TLE4 functions predominantly as a nuclear transcriptional co-repressor, the attenuated Co-IP signal reflects diminished nuclear TLE4–β-catenin complex formation and reduced β-catenin availability, rather than reduced TLE4 expression itself. Stimulus-induced nucleocytoplasmic redistribution has been described for other TLE family members (Brunquell et al., 2012; Zahavi et al., 2017), supporting the notion that quercetin-induced TLE4 relocalization may represent a regulated event contributing to its anti-tumor activity. Furthermore, we performed rescue experiments using the Wnt/β-catenin agonist one to determine whether quercetin-mediated Wnt inhibition is specifically mediated through the Wnt pathway (Figure 6G). Treatment with Wnt agonist one alone markedly upregulated β-catenin, Cyclin D1, and c-Myc, confirming activation of the Wnt pathway, whereas quercetin alone upregulated TLE4 and suppressed these targets. In the co-treatment group, although β-catenin levels were partially rescued compared with the quercetin-alone group, Cyclin D1 and c-Myc remained markedly suppressed. This result indicates that quercetin could effectively suppress downstream target gene expression even when β-catenin was partially rescued, suggesting that the inhibitory effect of quercetin on Wnt signaling may occur, at least in part, at the transcriptional level downstream of β-catenin. In addition, because TLE4 is involved in multiple signaling pathways including Notch and TGF-β (Turki-Judeh and Courey, 2012), the suppressive effects of quercetin on Cyclin D1 and c-Myc may be partially mediated through these pathways as well. Future studies using specific pathway inhibitors will help to further clarify the targets through which quercetin exerts its effects. Together with the observation that quercetin still suppressed migration and invasion in TLE4-knockout cells (Figures 8A–D), these findings indicate that TLE4 is an important, though not essential, mediator of quercetin-induced Wnt repression and anti-tumor activity.

The cell lines employed in this study exhibit distinct HPV infection statuses. High-risk HPV E6 and E7 oncoproteins have been extensively documented to manipulate multiple cellular signaling pathways, among which the Wnt/β-catenin pathway plays a particularly prominent role in HPV-associated cervical carcinogenesis (Muñoz-Bello et al., 2015; Peng et al., 2024). Mechanistically, HPV E6 activates canonical Wnt/β-catenin signaling through multiple convergent mechanisms: E6 promotes Wnt/β-catenin signaling via E6AP-mediated degradation of the negative regulator NHERF1 (Drews et al., 2019), which has been identified as a negative regulator of β-catenin signaling in cervical cancer (Wang et al., 2018); enhances TCF/β-catenin-mediated transcriptional activity in an E6AP-dependent manner, without altering β-catenin protein stability (Lichtig et al., 2010); and upregulates Wnt7b mRNA expression in host cells and their secreted extracellular vesicles, which subsequently activate β-catenin signaling in recipient cells and promote tumor angiogenesis (Qiu et al., 2020). Beyond E6, the HPV E7 oncoprotein also contributes to Wnt pathway activation: in HPV16-positive cells, E7 induces nuclear translocation of lactate dehydrogenase A (LDHA), where nuclear LDHA generates α-hydroxybutyrate to epigenetically activate antioxidant responses and Wnt signaling (Liu et al., 2018). Although HPV16 and HPV18 may adopt distinct upstream regulatory strategies, both subtypes converge on shared downstream Wnt-driven oncogenic programs (Peng et al., 2024). Notably, oncogenic activation of the Wnt/β-catenin pathway is not entirely dependent on sustained HPV oncogene expression. HPV-inactive cervical tumors, which have lost E6/E7 expression, exhibit elevated WNT/β-catenin signaling and are enriched for somatic mutations targeting WNT pathway genes, suggesting that Wnt activation serves as a convergent oncogenic mechanism irrespective of HPV status (Banister et al., 2017). Accordingly, these somatic genetic alterations confer constitutively enhanced Wnt signaling and proliferative potential in HPV-inactive cervical tumors. Nevertheless, our key findings—quercetin-induced TLE4 upregulation and subsequent suppression of Wnt/β-catenin signaling—were consistently detected in both HPV-positive and HPV-negative cervical cancer cells, indicating that this regulatory axis operates independently of HPV status, at least in the cell lines examined. The present study primarily focuses on the anti-cervical cancer mechanism of quercetin through TLE4-mediated Wnt pathway inactivation, and only verifies the regulatory effect of TLE4 on the Wnt pathway in different cervical cancer cell lines, without in-depth investigation into the specific interference of HPV infection status on this TLE4/Wnt molecular regulatory axis, which constitutes one of the limitations of this study.

Quercetin treatment effectively reversed the malignant phenotype and reduced the migration and invasion of TLE4-knockout Ca Ski cells, suggesting a functional compensatory effect for TLE4 deficiency. Further mechanistic investigation revealed that quercetin treatment markedly inhibited the Wnt/β-catenin signaling pathway activated by TLE4 knockout, as evidenced by the significant downregulation of key molecules of the Wnt/β-catenin signaling pathway and its downstream target genes. These findings clarify that quercetin reverses the enhanced migration and invasion abilities of cervical cancer cells induced by TLE4 knockout by inhibiting the aberrantly activated Wnt/β-catenin signaling pathway, thereby exerting its anti-tumor effect. The observation that, after quercetin treatment, the migration and invasion of TLE4-knockout cells remained higher than those of quercetin-treated wild-type cells indicates that the inhibitory effect of quercetin is partially dependent on TLE4 (Figures 8A–D). TLE4 therefore represents an important, though not essential, mediator of the anti-tumor effects of quercetin.

## 5 Conclusion

Through systematic screening, we identified TLE4 as a potential targeted therapeutic gene of quercetin. Experimental evidence confirmed that in cervical cancer, TLE4 may function as a tumor suppressor and serve as a key downstream molecule mediating the antitumor effects of quercetin. Further studies demonstrated that quercetin targets and upregulates TLE4 expression, thereby inhibiting the migration and invasion of cervical cancer by blocking the Wnt/β-catenin signaling pathway.

## Data Availability

The datasets generated during the current study are available in the BioProject repository, http://www.ncbi.nlm.nih.gov/bioproject/1377434. The datasets analysed during the current study are available in the GEO repository, https://www.ncbi.nlm.nih.gov/geo/.
