# Supplementary material for: Quercetin upregulates TLE4 to block the Wnt/β-catenin signaling pathway and inhibit cell migration and invasion of cervical cancer cells
Source: Front Pharmacol. 2026 Oct 7;17:1892382. doi: 10.3389/fphar.2026.1892382 (PMC13684866; doi:10.3389/fphar.2026.1892382)
Supplement: Supplementary file 1 [file DataSheet1.pdf]

A

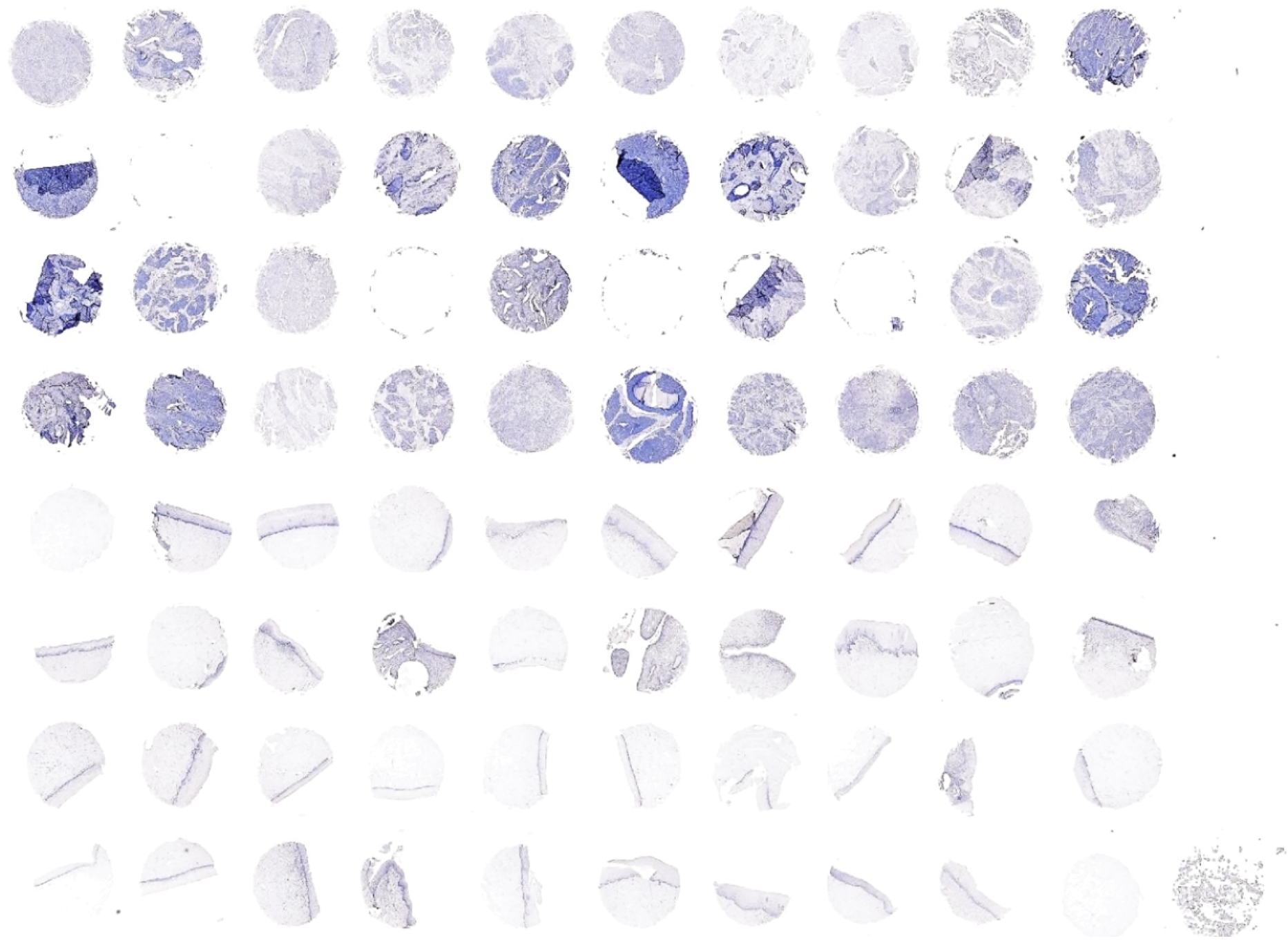

B

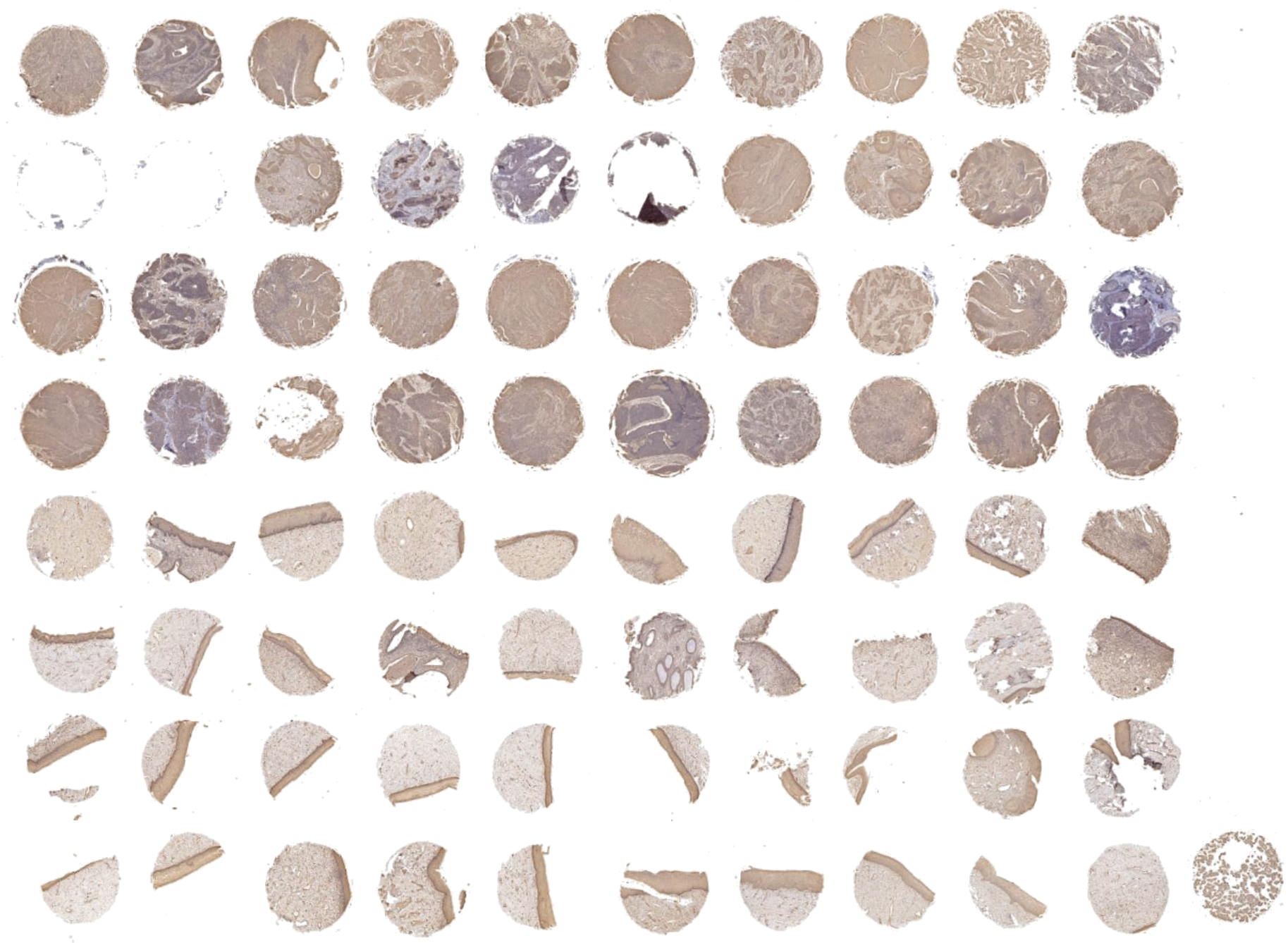

C

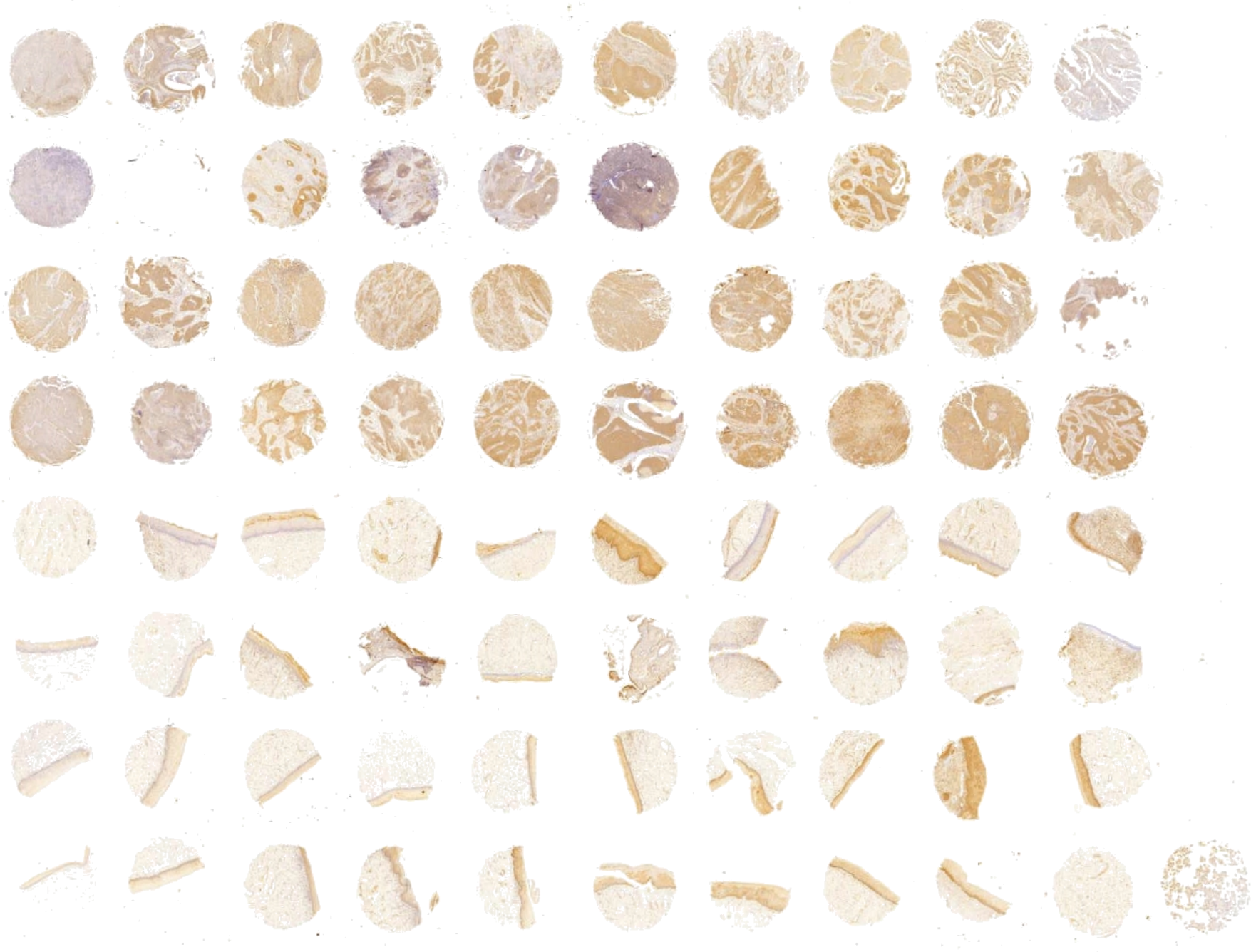

A

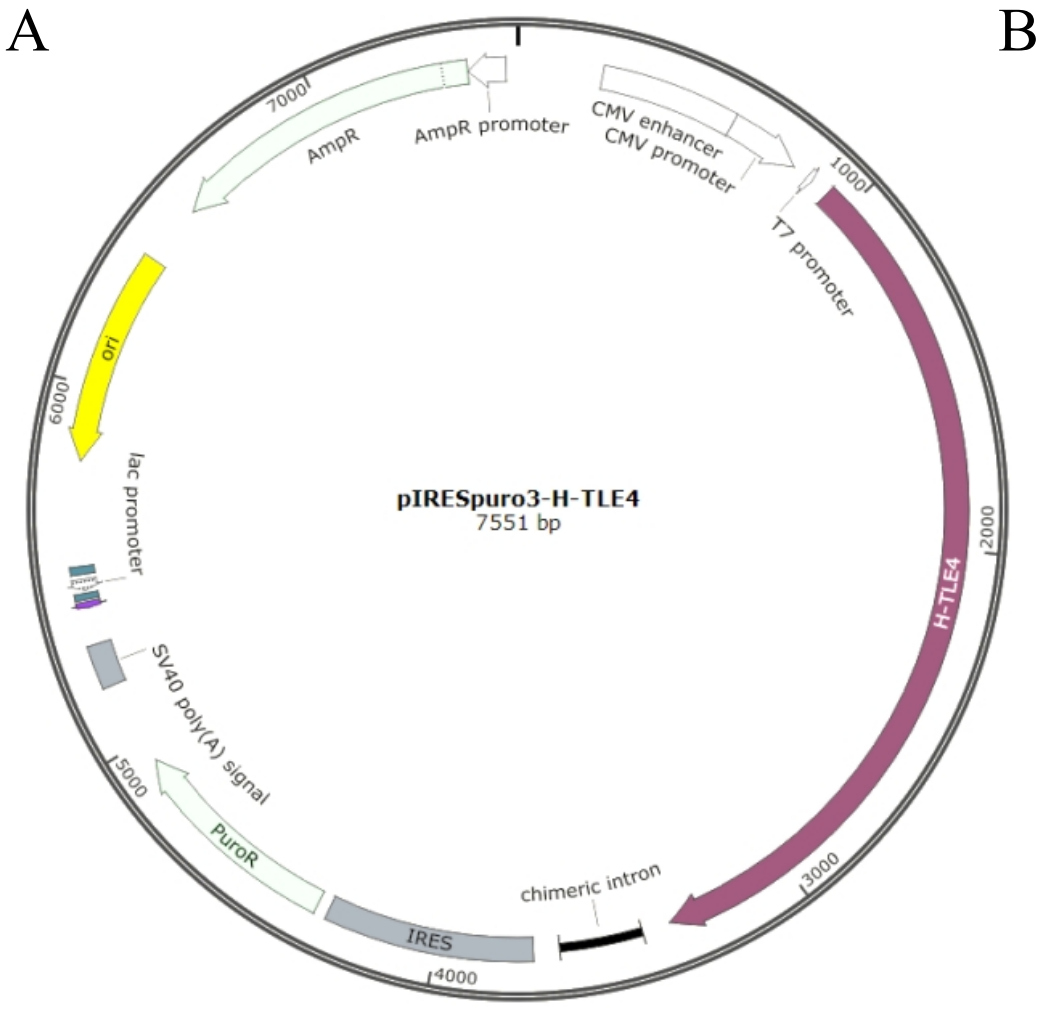
pIRESpuro3-H-TLE4
7551 bp
CMV enhancer
CMV promoter
T7 promoter
H-TLE4
chimeric intron
IRES
PuroR
SV40 poly(A) signal
lac promoter
ori
AmpR
AmpR promoter
1000
2000
3000
4000
5000
6000
7000

B

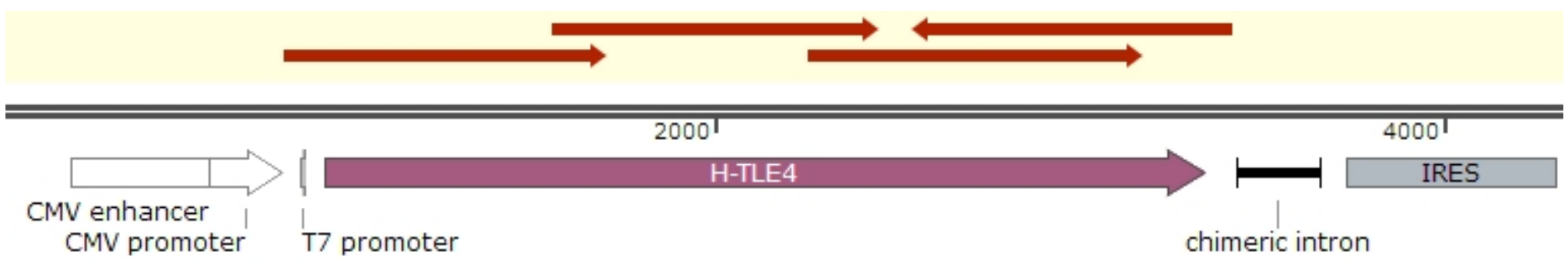
2000
4000
H-TLE4
IRES
CMV enhancer
CMV promoter
T7 promoter
chimeric intron

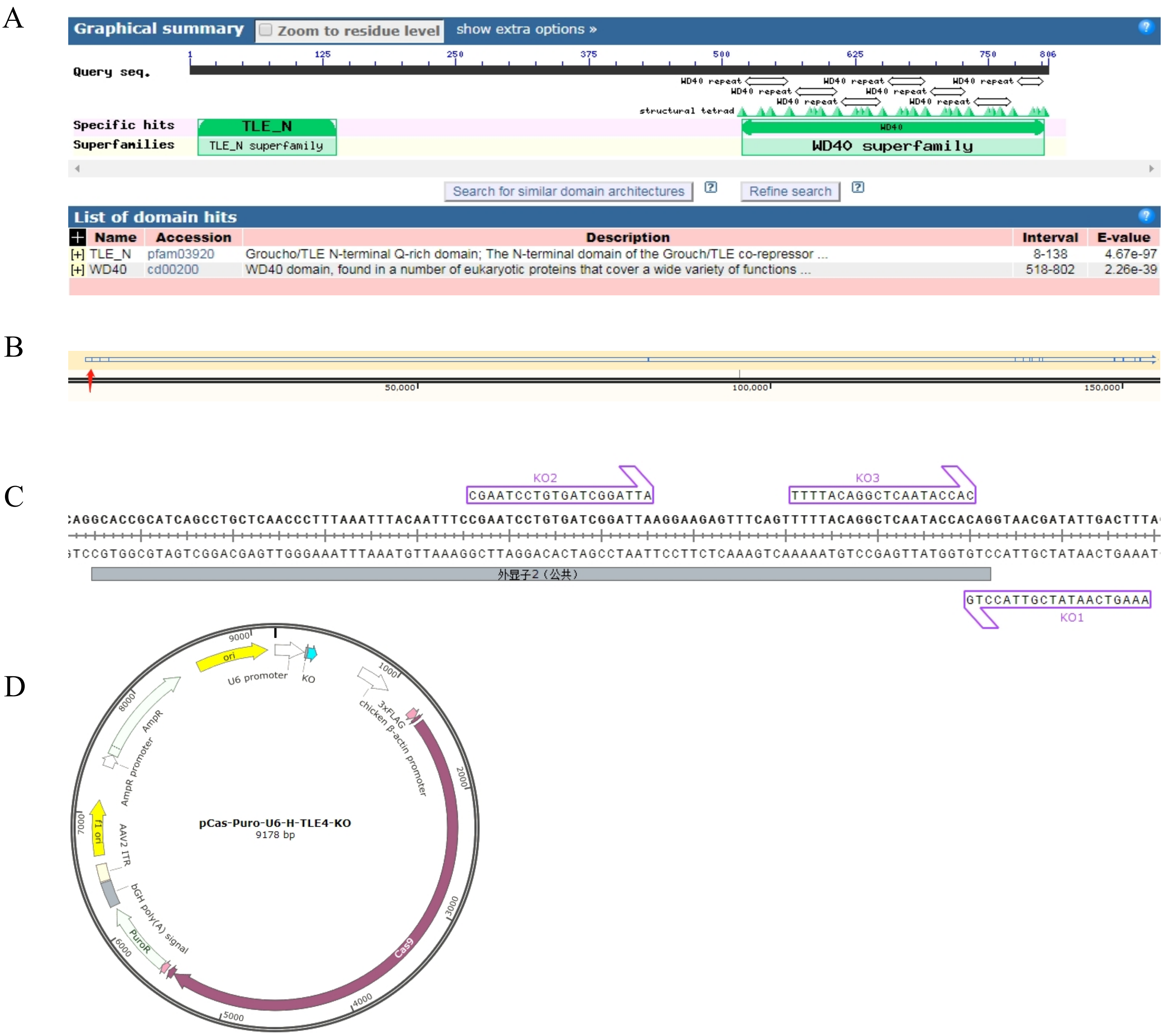
A
Graphical summary
Zoom to residue level
show extra options »
Query seq.
1
125
250
375
500
625
750
806
WD40 repeat
structural tetrad
Specific hits
Superfamilies
TLE_N
TLE_N superfamily
WD40
WD40 superfamily
Search for similar domain architectures
Refine search
List of domain hits
Name
Accession
Description
Interval
E-value
[+] TLE_N pfam03920 Groucho/TLE N-terminal Q-rich domain; The N-terminal domain of the Grouch/TLE co-repressor ... 8-138 4.67e-97
[+] WD40 cd00200 WD40 domain, found in a number of eukaryotic proteins that cover a wide variety of functions ... 518-802 2.26e-39
B
50,000
100,000
150,000
C
KO2
CGAATCCTGTGATCGGATTA
KO3
TTTTACAGGCTCAATACCAC
AGGCACCGCATCAGCCTGCTCAACCCTTTAAATTTACAATTTCCGAATCCTGTGATCGGATTAAGGAAGAGTTTCAGTTTTTACAGGCTCAATACCACAGGTAACGATATTGACTTTA
TCCGTGGCGTAGTCGGACGAGTTGGGAAATTTAAATGTTAAAGGCTTAGGACACTAGCCTAATTCCTTCTCAAAGTCAAAAATGTCCGAGTTATGGTGTCCATTGCTATAACTGAAAT
外显子2（公共）
GTCCATTGCTATAACTGAAA
KO1
D
pCas-Puro-U6-H-TLE4-KO
9178 bp
ori
U6 promoter
KO
3xFLAG
chicken β-actin promoter
Cas9
bGH poly(A) signal
PuroR
AAV2 ITR
f1 ori
AmpR promoter
AmpR
1000
2000
3000
4000
5000
6000
7000
8000
9000

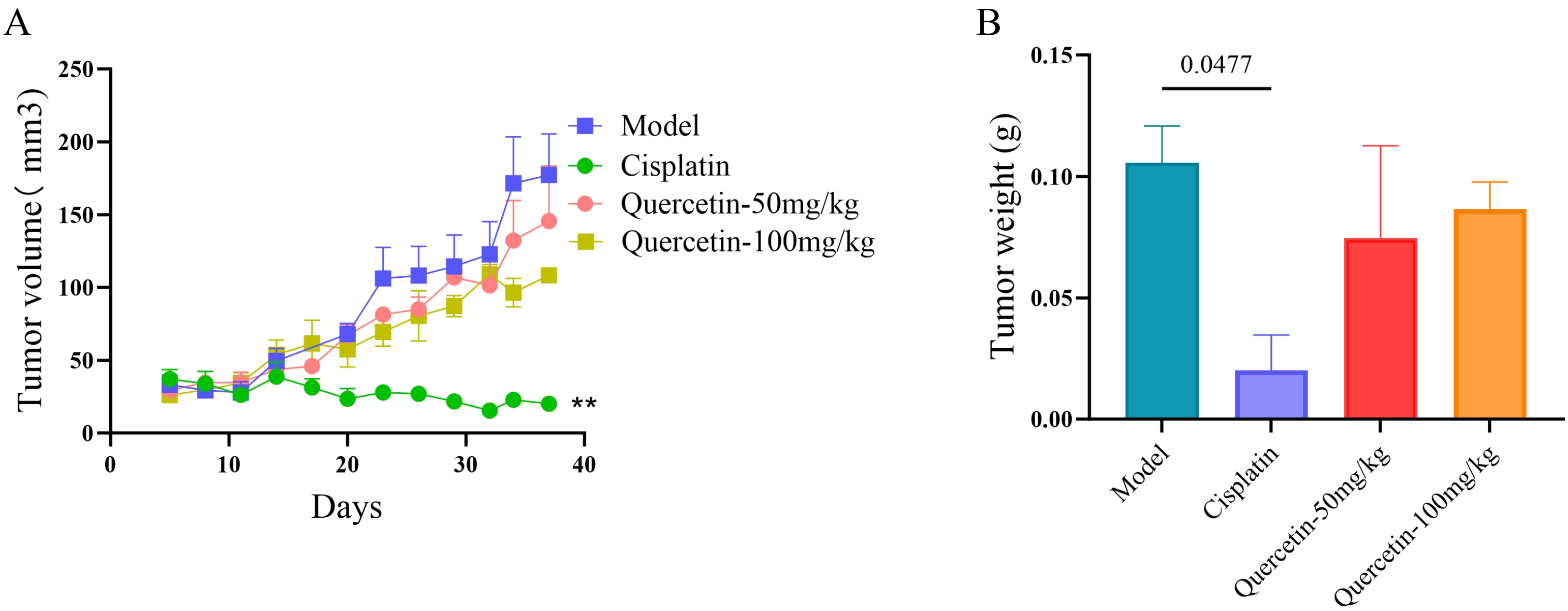

A
Tumor volume(mm3)
250
200
150
100
50
0
0
10
20
30
40
Days
Model
Cisplatin
Quercetin-50mg/kg
Quercetin-100mg/kg
**
B
Tumor weight (g)
0.15
0.10
0.05
0.00
0.0477
Model
Cisplatin
Quercetin-50mg/kg
Quercetin-100mg/kg

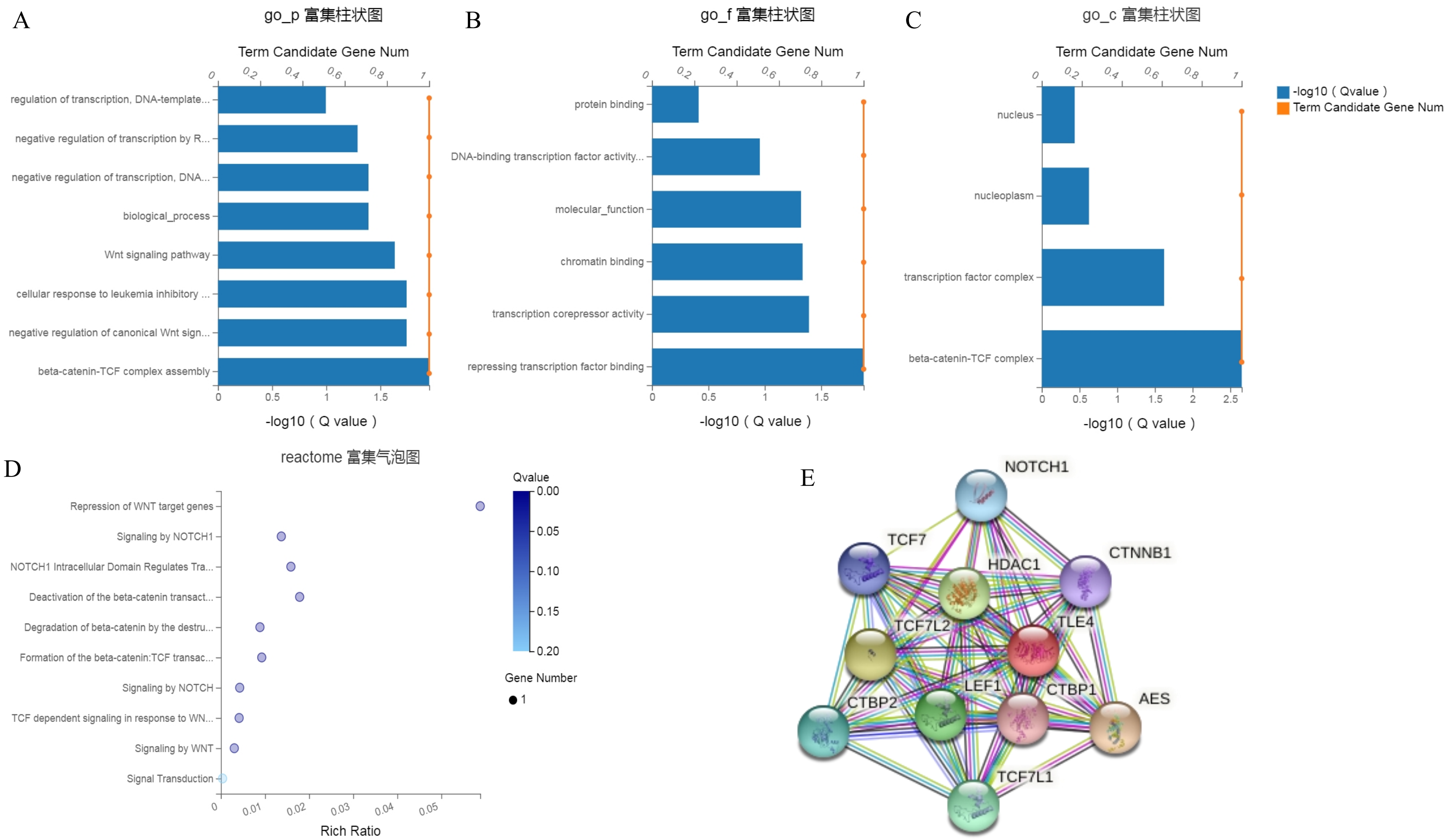

A
go_p 富集柱状图
Term Candidate Gene Num
regulation of transcription, DNA-template...
negative regulation of transcription by R...
negative regulation of transcription, DNA...
biological_process
Wnt signaling pathway
cellular response to leukemia inhibitory ...
negative regulation of canonical Wnt sign...
beta-catenin-TCF complex assembly
-log10 (Q value)
B
go_f 富集柱状图
Term Candidate Gene Num
protein binding
DNA-binding transcription factor activity...
molecular_function
chromatin binding
transcription corepressor activity
repressing transcription factor binding
-log10 (Q value)
C
go_c 富集柱状图
Term Candidate Gene Num
nucleus
nucleoplasm
transcription factor complex
beta-catenin-TCF complex
-log10 (Q value)
-log10 (Qvalue)
Term Candidate Gene Num
D
reactome 富集气泡图
Repression of WNT target genes
Signaling by NOTCH1
NOTCH1 Intracellular Domain Regulates Tra...
Deactivation of the beta-catenin transact...
Degradation of beta-catenin by the destru...
Formation of the beta-catenin:TCF transac...
Signaling by NOTCH
TCF dependent signaling in response to WN...
Signaling by WNT
Signal Transduction
Rich Ratio
Qvalue
Gene Number
E
NOTCH1
TCF7
HDAC1
CTNNB1
TCF7L2
TLE4
LEF1
CTBP2
CTBP1
AES
TCF7L1
